# A species-wide inventory of receptor-like kinases in *Arabidopsis thaliana*

**DOI:** 10.1186/s12915-025-02364-y

**Published:** 2025-08-26

**Authors:** Zachary Kileeg, G. Adam Mott

**Affiliations:** 1 Department of Biological Sciences, University of Toronto - Scarborough, Toronto, Canada; 2 Department of Cell and Systems Biology, University of Toronto, Toronto, Canada; 3 Centre for the Analysis of Genome Evolution & Function, University of Toronto, Toronto, Canada

**Keywords:** Pan-RLKome, RLKs, RLK evolution, Plant immunity, PTI

## Abstract

**Background:**

The receptor-like kinases (RLKs) are the largest family of proteins in plants. Characterized members play critical roles in diverse processes from growth to immunity, and yet the majority do not have a known function. Assigning function to RLKs poses a significant challenge due to the specificity of ligand recognition and because of the often pleiotropic or redundant functions RLKs possess. These problems inhibit the important work of identifying stress-related receptors that may be targets for crop improvement. Identification of stress-related evolutionary signatures can provide a way to expedite the discovery of candidate receptors. Pan-genome analysis can be used to compare naturally occurring variants within a species to identify evolutionary signatures that may otherwise be hidden by using only a single ecotype.

**Results:**

Using 146 ecotypes of Arabidopsis, we generated a pan-RLKome to investigate species-wide natural diversity and identify structural variation and other patterns indicative of stress adaptation. We discovered significant presence/absence variation across a subset of RLKs, most of which occurred in specific subclades nested within receptor subfamilies. These same subclades tended to have arisen through proximal or tandem duplication, both of which are common mechanisms during the expansion of stress-related genes. We also identified strong positive selection across many gene subfamilies and a bias of positive selection in the extracellular domains of receptors. This suggests escape from adaptive conflict within the extracellular domain may have played a large role in the evolution and adaptation of the RLKs.

**Conclusion:**

Taken together, this work represents an excellent tool for the comparative study of RLKs and has identified lineages and subclades within RLK subfamilies with the hallmarks of involvement in stress adaptation.

**Supplementary Information:**

The online version contains supplementary material available at 10.1186/s12915-025-02364-y.

## Background

Plants use a vast network of cell surface receptors to recognize and respond to a variety of signals to optimize fitness and control critical biological processes ranging from growth to immune signaling [1–3]. In *Arabidopsis thaliana* (hereafter Arabidopsis)*,* there are over 600 receptor-like kinases (RLKs) that perceive and integrate signals [1, 4]. The RLKs can be divided into membrane-localized receptors and membrane-interacting cytoplasmic kinases [2, 3]. The membrane-localized RLKs consist of a semi-conserved intracellular kinase domain (KD), a single-pass transmembrane region, and an extracellular domain (ECD) with varying domain architectures [1, 4]. The RLKs have been separated into 15 different subfamilies based on the conservation of the kinase domain [4, 5]. Each subfamily displays a single type of ECD from the diversity seen in RLKs generally [4]. In spite of this structural relatedness, subfamily members often have disparate biological functions [3, 4].

The largest subfamily of RLKs is the leucine-rich repeat receptor-like kinases (LRR-RLK), which is characterized by an ECD containing a number of leucine-rich repeats [4, 6, 7]. The approximately 225 LRR-RLKs have been separated into 15–24 subfamilies, and individual members have been shown to play important roles in processes ranging from development to immunity [3, 5, 8, 9]. The LRR-RLKs also contain three structurally divergent subclades (LRR-RLK-I, LRR-RLK-VIII.1, and LRR-RLK-VIII.2) that contain sugar-binding malectin or malectin-like domains in their ECDs in addition to LRR repeats [4, 7]. Together, these three subclades form a paraphyletic group we will refer to as the malectin-containing LRRs (MLRRs). Individual members of these receptor subclades have been linked to biotic stress responses and recognition of pollen [10–14].

Many other RLK subfamilies may act through cell-wall sensing. For example, the cysteine-rich repeat RLK (CRK) family contributes to both abiotic and biotic responses possibly through cell-wall sensing [4, 15, 16]. The epidermal growth-factor (EGF) containing wall-associated kinases (WAKs) and WAK-like (WAKL) receptors (hereafter referred to together as WAKLs) and the structurally related LRK10-like receptor-like kinases (LRK10L) have been implicated in protection from a variety of fungal and bacterial pathogens [1, 3, 4, 17–19]. The malectin-containing *Catharanthus roseus* receptor-like kinase 1-Like (CrRLK1L) subfamily has also been implicated in cell wall integrity sensing, defense responses, and mediation of development and reproductive responses [1, 3, 4]. The crinkly4-like (CR4L) family members have partially redundant roles that relate to growth and development in a cell wall related manner [1, 20, 21]. Lastly, the proline-rich extensin-like receptor-like kinases (PERKs) are widely distributed across plant species and are believed to contribute to root abiotic stress tolerance and growth control, possibly in a cell wall related manner [1, 22].

Another prevalent domain across RLK subfamilies is the lectin domain [4, 23]. L-type lectin RLKs (LecRLKs) contribute to immune signaling through recognition of microbial or damage signals [23, 24]. However, no known LecRLKs have been shown to bind carbohydrates [24]. The G-type are often found alongside an S-locus in the S-locus RLKs (SRKs), contributing to self- and non-self compatibility as well as biotic stress responses [1, 23, 24]. The lysin-motif containing RLKs (LRKs) can bind various carbohydrates but are most known for recognition of chitin [1, 3, 24]. They include the commonly studied chitin elicitor receptor-like kinase 1 (CERK1) and are important in recognition and response to fungal and bacterial infection [1, 24].

Not all RLKs are membrane-anchored. The receptor-like cytoplasmic kinases (RLCKs) are a subfamily of RLKs lacking an ECD and mainly comprised of a cytoplasmic kinase domain, as most also lack a transmembrane domain [4, 25]. The RLCKs are a large and diverse subfamily separated into approximately 17 subclades [25].

Many of the RLKs that contribute to immune function do so by acting as pattern recognition receptors. Pattern recognition receptors are located at the cell surface and can recognize conserved microbial molecular patterns or damage-associated signals to activate pattern-triggered immunity [26, 27]. This system is reinforced through the activity of intracellular receptors known as the nucleotide-binding leucine-rich repeat receptors (NLRs) that recognize injected pathogen effectors and induce a more robust plant immune state [26, 27] While immune-related RLKs are attractive targets for crop improvement, their identification is often low-throughput, time-consuming, and costly. Searching for evolutionary markers of adaptation may be one way to identify candidate immune-related or stress-responsive receptors at scale.

Duplication due to structural rearrangement is one such marker associated with genes involved in stress response, but identifying this variation is difficult using only short-read generated genomes [28, 29]. The profusion of long-read sequencing technology and construction of pan-genomes in recent years has revealed previously hidden complex structural variation events including copy number variation (CNV) and presence/absence variation (PAV) [28, 30]. CNV and PAV, both of which often arise through tandem (TD) or proximal (PD) duplication events, are especially important mechanisms of stress adaptation [31–34].

A pan-genome is the collection of all potential genes found across the different strains or isolates of a given organism [35]. They have traditionally been used to investigate the changing genetic landscape in prokaryotes, but the development of cost-effective long-read sequencing has made their use possible in eukaryotes [28]. Currently, there are pan-genomes available for numerous agronomically important species including rice, barley, *Brassica oleracea, Brassica napus,* tomato, soybean, wheat, and grass [30, 36–42], as well as for the model organism Arabidopsis [43–46]. In these studies, pan-genome analysis has been successfully employed to discover agronomically important traits through identification of novel structural variation [30, 36–46]. Stress-adaptation, especially in response to pathogens, can result in rapid gene gain/loss and functionalization [27, 47]. Pan-genomes are especially useful in the identification of these signatures through investigation of biotic-stress related gene subfamilies [48–50]. A recent report used a modified pan-genome approach to catalogue the genetic variation of the stress-related NLRs across Arabidopsis [47]. The researchers generated a pan-NLRome using 64 accessions of Arabidopsis and showed extensive gene PAV and CNV, highlighting the necessity of such approaches to capture the diversity of stress-related genes in particular [47].

The RLKs are a popular gene family for study due to their roles in many agronomically important processes, yet most still have no known function. In this study, we use 146 ecotypes of Arabidopsis to investigate the natural diversity of RLKs and create a pan-RLKome. The analysis of this RLK inventory shows evidence of subclade-specific expansive gene gain/loss via TD and PD. We also show that the ECDs of receptors have more positive selection than the KDs, suggesting that the ECD is under selective pressure to diversify recognition. The results here have important implications for identifying subfamilies that may contain genes involved in stress adaptation and paint a picture of RLK adaptation and evolution across Arabidopsis.

## Results

### Samples and gene discovery

**Fig. 1:**
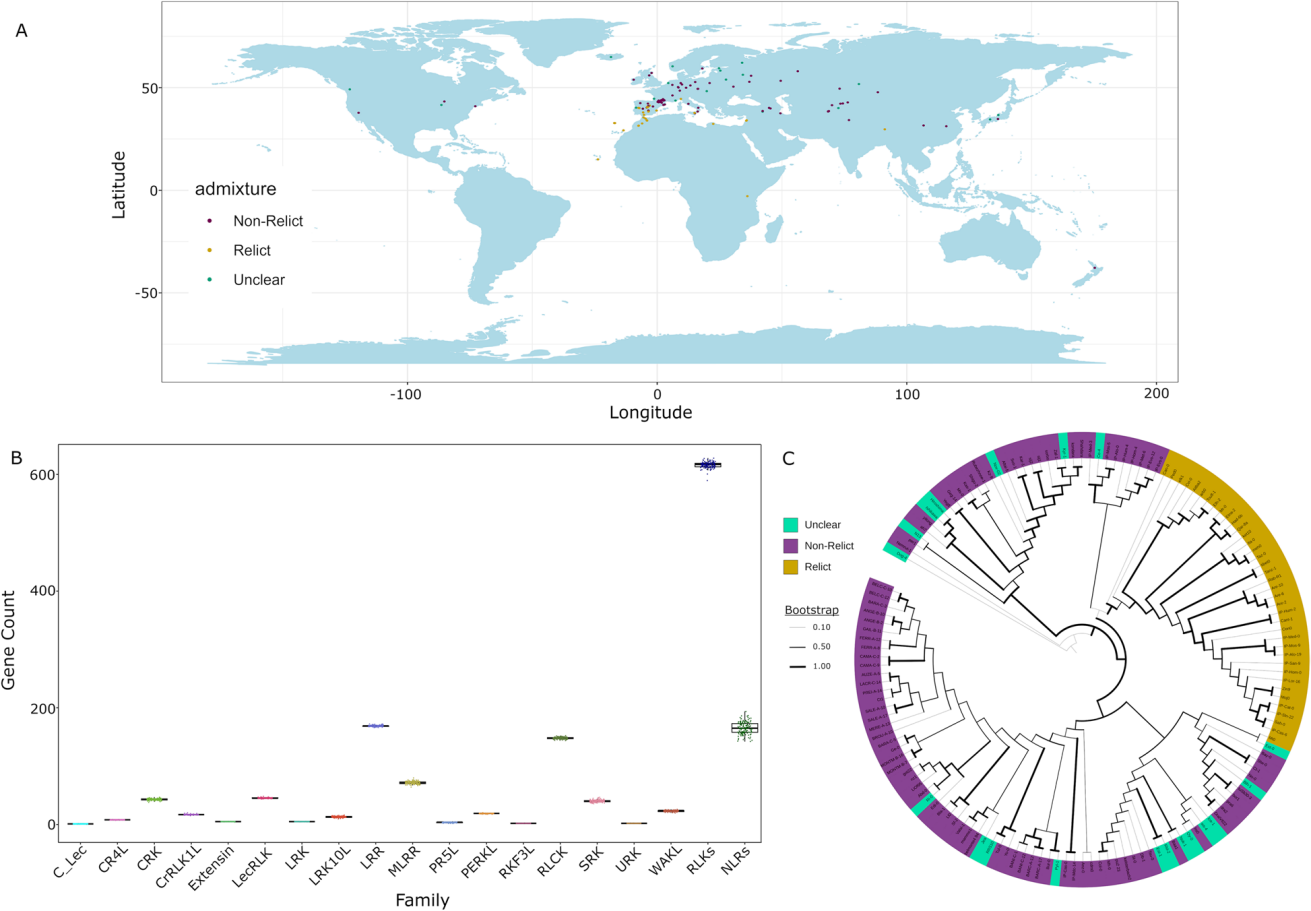
Ecotypes show minimal variation in gene counts per gene subfamily. **A**. Ecotypes used in this study are mapped based on the latitude and longitude where that ecotype was collected. Ecotype admixture group is coloured for non-relict (purple), relict (yellow), and populations where the grouping is unclear (teal). **B**. Boxplot of the number of genes per gene subfamily for RLKs and NLRs across 146 ecotypes. **C**. Species tree based on the single copy OGs for RLKs output from orthofinder. Ecotypes are coloured based on their admixture group for non-relict (purple), relict (yellow), and populations where the grouping is unclear (teal). Line thickness represents bootstrap support from 1000 replicates with thicker = higher support

To explore the diversity of RLK sequences in Arabidopsis, we sought to compile a species-wide gene inventory of all RLKs and create a pan-RLKome. We searched public sources and downloaded all genome sequences for 146 Arabidopsis ecotypes from four sources (Additional file 1: Supplemental Table 1) [44–46, 51]. The available sequences represent a broad collection of ecotypes including both relicts and non-relicts from across the globe (Fig. 1A). For each ecotype, we predicted protein sequences using a combination of homology, de novo, and transcript prediction methods. To identify RLKs, we first identified all kinase-containing proteins in each genome, clustered these genes into orthogroups (OGs), and performed pairwise comparisons of each OG to the Araport11 protein set and filtered out any OGs where the best hit was not an annotated RLK. For NLR discovery, we used Resistify [52]. Each ecotype had over 600 RLK sequences and approximately 165 NLR sequences (Fig. 1B). The number of RLKs found in each subfamily varied slightly between ecotypes (Fig. 1B).

**Table 1:** Top 20 OGs with the highest average gene-wide non-synonymous to synonymous substitution ratios

Orthogroup	Most similar gene	Nonsyn/syn ratio	Family	Pagenome	Duplication mechanism
N0.HOG0001086	AT3G45860/CRK4	1.68381145	CRK	Cloud	Proximal
N0.HOG0001056_1	AT1G29720/RKFL1	1.14189715	MLRR	Shell	Tandem
N0.HOG0000002_3	AT1G65250	1.13743683	RLCK	Cloud	Proximal
N0.HOG0001064	AT4G11460/CRK30	0.9750583	CRK	Shell	Tandem
N0.HOG0001088	AT1G17910	0.96804872	WAKL	Cloud	Dispersed
N0.HOG0001076_3	AT4G21380/RK3	0.96201449	SRK	Cloud	Proximal
N0.HOG0000039_3	AT1G35710	0.95987088	LRR	Cloud	Proximal
N0.HOG0001080	AT3G25490	0.93436267	WAKL	Cloud	Tandem
N0.HOG0000002_2	AT1G65250/ZRK14	0.92794967	RLCK	Shell	Proximal
N0.HOG0000024	AT5G59680	0.92643537	MLRR	Cloud	Proximal
N0.HOG0000047_2	AT5G07280/EMS1	0.92611325	LRR	Cloud	Proximal
N0.HOG0000006	AT1G65250	0.92258907	RLCK	Cloud	Proximal
N0.HOG0001072	AT5G65600/LecRK-IX.2	0.91904901	LecRLK	Cloud	Dispersed
N0.HOG0000011	AT2G14510	0.90857451	MLRR	Cloud	Dispersed
N0.HOG0001104	AT5G39030/MDS4	0.90762346	CrRLK1L	Core	Tandem
N0.HOG0000990	AT2G30730/CARK9	0.90597381	RLCK	Almost Core	WGD/Segmental
N0.HOG0001094	AT4G23300/CRK22	0.90355009	CRK	Cloud	Dispersed
N0.HOG0001108	AT3G45390/LecRK-I.2	0.88953295	LecRLK	Core	WGD/Segmental
N0.HOG0001083	AT3G57710/ZRK1	0.88329522	RLCK	Cloud	Tandem
N0.HOG0000038_2	AT1G61390	0.88010636	SRK	Cloud	Tandem

### A species-wide RLK gene inventory

To investigate the variation in RLK gene content across and between ecotypes, we used the OGs of RLKs identified according to their sequence similarity. A similar analysis of the NLRs as a benchmark of a diverse, biotic-stress associated receptor family was also conducted. Using the single-copy OGs, we then inferred the phylogenetic relationship of ecotypes. Ecotypes tended to form clades clustering those isolated from geographically similar regions, with relicts and non-relicts clustering among themselves (Fig. 1C). The only exception is the relict ecotype Mt-0, which clustered together with non-relicts (Fig. 1C).

**Fig. 2:**
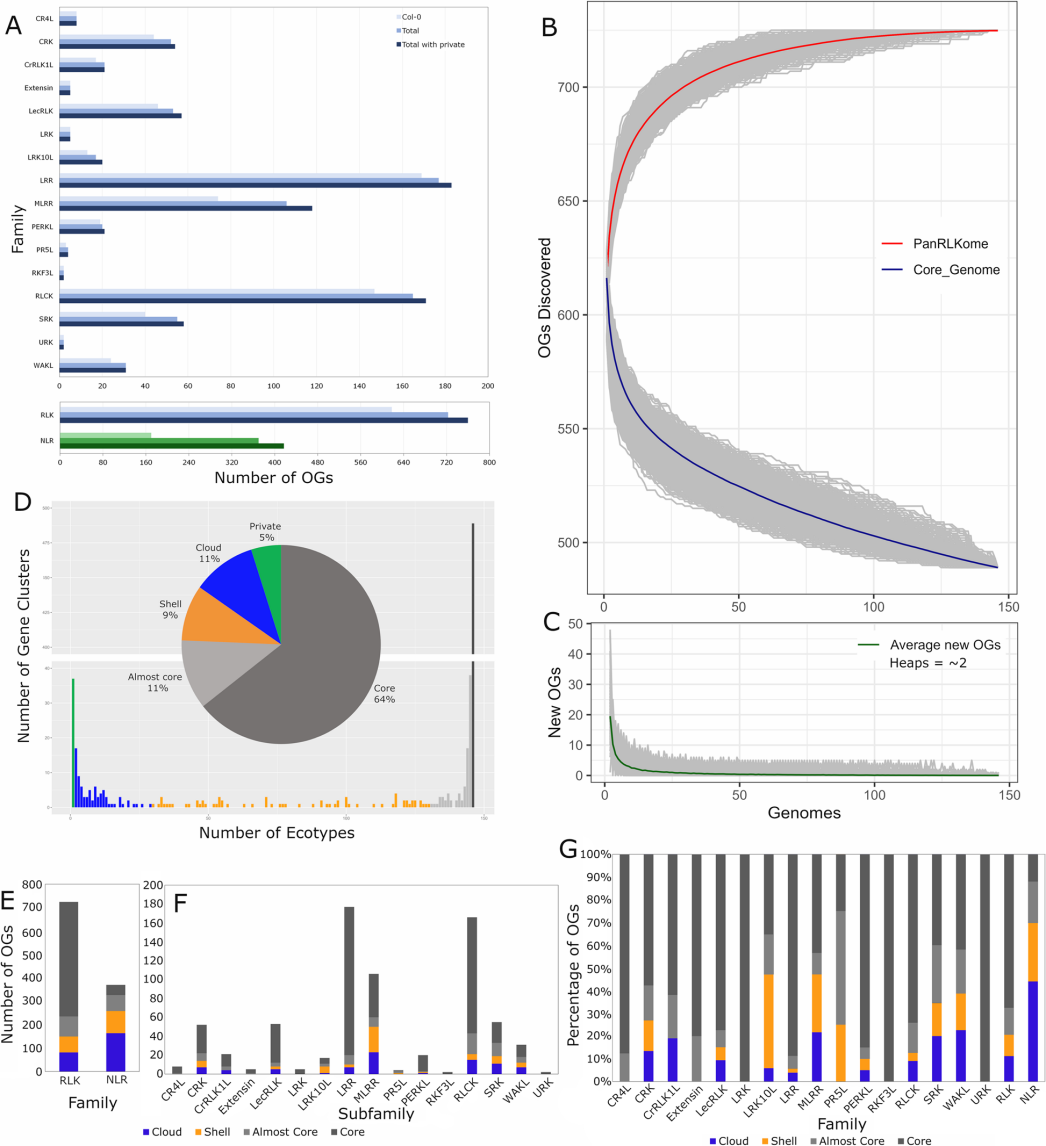
Orthogroup distribution and saturation. **A**. Number of genes or OGs from each RLK subfamily or NLR family in Col-0, across all ecotypes, and across all ecotypes including private (single accession) genes. **B**-**C**. Discovery of novel OGs and pan-RLKome saturation. Rarefaction (**B**) and collector’s (**C**) curves show average number of core OGs and newly discovered OGs per ecotype added respectively. Order of ecotype addition for both analyses was randomized 1000 times and averages plotted. **D**. Distribution and diversification of OGs. OGs were assigned to core, almost core, shell, cloud, and private sets based on the number of ecotypes in which they were found. 75% of OGs are core or almost core, while 25% of OGs are dispensable. **E**-**G**. RLKs were split into counts by family (**E**), by RLK subfamily (**F**), or by proportion of RLKs (**G**). In **E** and **F**, the total number of OGs found in each family or subfamily were coloured by pan-genome category and counted for each. In **G**, the proportion of OGs found in each pan-genome category was calculated for each subfamily. The NLRs were added as a benchmark to both **E** and **G**

The RLKs have been assigned to subfamilies, each of which carries a characteristic ECD structure [4, 5]. To determine the distribution and pan-genome category of the different RLK OGs, we took the inferred phylogenetic hierarchical OGs from OrthoFinder, split over-clustered OGs into smaller orthogroups, and assigned each OG to its respective subfamily based on the most similar annotated RLK. Overall, there are approximately 723 OGs present in at least two different ecotypes found within the pan-RLKome (Fig. 2A). The pan-RLKome size including private genes (those found in only one ecotype) is approximately 760 OGs (Fig. 2A). The Col-0 genome alone contains 618 RLKs, while the addition of 145 ecotype genomes identified 106 new OGs (142 OGs including private genes) (Fig. 2A). The pan-RLKome contains additional OGs when compared to Col-0 alone for the majority of subfamilies (Fig. 2A). The MLRR, RLCK, and SRK subfamilies show the largest diversity with the greatest number of novel OGs not found in Col-0 (Fig. 2A). The LecRLK, LRK10L, LRR, and WAKL subfamilies also see minor increases, while the extensin, LRK, RKF3L, and URK subfamilies see no novel OGs (Fig. 2A).

Consistent with previous reports, the NLRs show much larger variability [47]. Across all ecotypes, the pan-NLRome has over double the number of OGs that are found in Col-0 (Fig. 2A). When we include private genes, the approximate number of NLR OGs rises to over 400 from the 170 present in Col-0 (Fig. 2A).

When we looked at the accumulation of OGs across ecotypes, the inventory begins to saturate after the addition of only ~ 10–25 ecotypes with just over 20 new OGs discovered after the addition of the remaining ~ 120 ecotypes (Fig. 2B, C). After the addition of approximately 10 ecotypes, we see less than 5 new OGs per added ecotype (Fig. 2C). After 100 added ecotypes, the number of new OGs found is less than one per ecotype with a final rate of ~ 0.75 new OG per ecotype after the addition of 146 genomes (Fig. 2C). These numbers vary only slightly with the inclusion of private genes (Additional file 2: Supplemental Fig. 1A, B). In both cases, the Heap’s estimated alpha is ~ 2, indicating the pan-RLKome is closed (Fig. 2C, Additional file 2: Fig. S1B).

To investigate the distribution of OGs across ecotypes, we counted the number of ecotypes that contained an orthologue within a given OG and separated the OGs into core (present in 146 ecotypes), almost core (131–145 ecotypes), shell (30–130 ecotypes), cloud (2–29 ecotypes), and private genomes (1 ecotype). The core RLK set is composed of ~ 480 OGs (Fig. 2B), and ~ 75% of OGs are contained within the core or almost core genomes, with a further ~ 20% within the shell and cloud genomes (Fig. 2D). The final ~ 5% are contained within the private genome, but were not included in further analyses as they may be the result of misannotation. As a benchmark for a stress-related receptor family, we performed the same analysis on the NLRs. In contrast to the RLK distribution, the NLRs have many more OGs contained within the dispensable genome, and fewer in the core and almost core (Fig. 2E). The NLRs contain almost twice as many OGs within the dispensable genome compared to the RLKs (~ 258 to ~ 143) (Fig. 2E). Among the RLK subfamilies, the MLRRs have the highest number of OGs within the dispensable genome (~ 50), while the RLCKs and SRKs both have more than 20 (Fig. 2F). If we instead measure proportions, the NLRs still contain a much higher proportion of their OGs within the dispensable genome compared to the RLKs (~ 70% to ~ 20%) (Fig. 2G). Among the RLKs, the LRK10L and MLRR subfamilies have the largest proportion of OGs within the dispensable genome with just under 50% (Fig. 2G). The CRK, PR5L, SRK, and WAKL subfamilies contain over 20% of OGs within the dispensable genome, followed by the CrRLK1Ls, LecRLKs, and RLCKs which contain over 10% (Fig. 2G).

### RLK subfamilies show high rates of tandem and proximal duplication

**Fig. 3:**
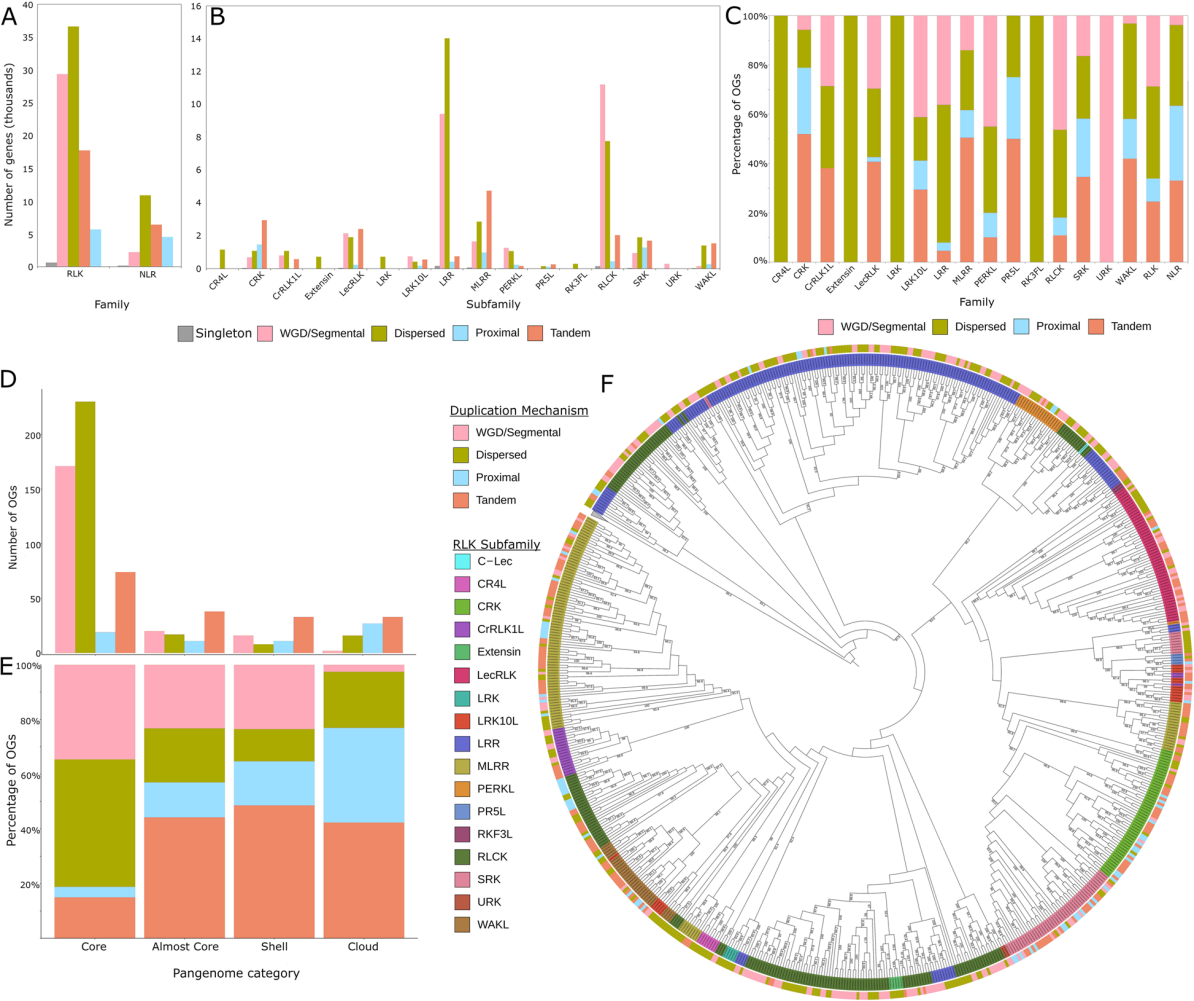
Mechanism of duplication and phylogeny of RLKs. **A**-**B**. Total number of genes across all 146 ecotypes that arose through dispersed, proximal, tandem, or WGD/Segmental duplication, or are singletons divided by subfamily. Mechanism of duplication was inferred using MCScanX. **C**. Proportion of NLR and RLK subfamily OGs in which the majority of members in that OG arose through a given duplication mechanism. **D**-**E**. The total number in **D** or proportion in **E** of RLK core, almost core, shell, and cloud OGs that arose by a specific duplication mechanism. **F**. Phylogeny inferred from consensus sequences of the RLK kinase domains for each OG. Each node represents an OG. The representative sequence for each OG is the consensus kinase domain of all genes in that OG. Bootstrap values represent 1000 replicates. The inner circle labels correspond to the RLK family assigned to the most closely related annotated gene in Col-0. The outer circle represents the duplication mechanism by which the majority of genes in that OG arose

Duplication followed by neofunctionalization is an important driver of gene functional divergence [53, 54]. It is therefore not surprising that genes that have arisen via tandem or proximal duplication are over-represented in genes involved in stress responses [31, 34]. This observation led us to investigate the mechanism of duplication responsible for RLK and NLR expansion. The most common duplication mechanism in the RLKs is dispersed duplication (DSD), followed by whole genome/segmental duplication (WGD/Segmental), tandem duplication (TD), proximal duplication (PD), and lastly singletons (Fig. 3A). Within the NLRs, DSD is also the most common mechanism of duplication, followed by TD, PD, WGD/Segmental, and lastly singleton (Fig. 3A). We also examined the frequency of duplication mechanisms within each subfamily of RLKs. Across the different RLK subfamilies, TD was the most common mechanism of duplication for the CRK, LecRLK, MLRR, PR5L, and WAKL subfamilies (Fig. 3B). The SRKs display a similar distribution of mechanisms to that observed in the NLRs (Fig. 3B).

While NLRs overall display a higher proportion of OGs that have arisen by PD or TD, a subset of RLK subfamilies shows a similar pattern of PD or TD occurrence (Fig. 3C). The CRK, MLRR, PR5L, SRK, and WAKL subfamilies all show elevated rates (≥ 50%) of OGs that arose through TD or PD, while the CRK and PR5L subfamilies have a higher proportion than seen in the NLRs (Fig. 3C). The LRK10L and LecRLK subfamilies also have a relatively high proportion, with over 40% of their OGs having arisen through TD or PD (Fig. 3C). The LRR, PERKL, and RLCK subfamilies show low rates of OGs that have arisen through TD or PD (< 20%) (Fig. 3C). Lastly, the CR4L, Extensin, LRK, RKF3L, and URK subfamilies have no TD or PD (Fig. 3A–C).

We next examined which duplication mechanism was the major driver of duplication for RLKs in each pan-genome category (Fig. 3D, E). For core OGs, the most common duplication mechanisms were WGD/Segmental and DSD which together accounted for approximately 80% of all OGs (Fig. 3D, E). The remaining ~ 20% of core OGs arose through TD and PD (Fig. 3D, E). The almost core, shell, and cloud OGs showed the opposite trend, where the majority of OGs arose through TD or PD (Fig. 3D, E). Cloud genes overall had the most OGs that arose through TD and the fewest that arose through WGD/Segmental duplication (Fig. 3D, E).

To examine how the different modes of duplication have affected the expansion of RLK subfamilies, we inferred a maximum likelihood phylogeny based on the KD consensus sequence for each RLK OG and labelled each representative OG by its most similar RLK subfamily and mechanism of duplication (Fig. 3F). OGs tended to cluster together based on their subfamily designation as previously annotated [4, 5] with high bootstrap support (> 90%; Fig. 3F).

### Rate of positive selection differs by pan-genome category and duplication mechanism

**Fig. 4:**
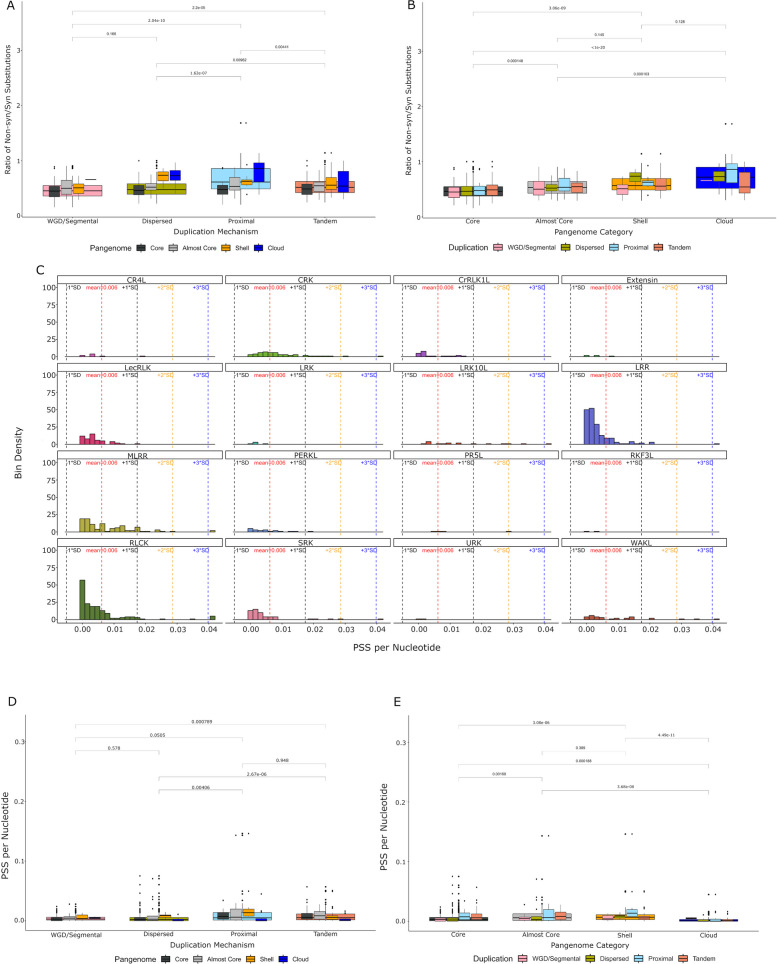
Rates of non-synonymous to synonymous substitutions and positive selection across orthologues. **A**-**B**. Boxes show the ratio of non-synonymous to synonymous substitutions gene-wide in each OG split by duplication mechanism in **A** or pan-genome category in **B**. Boxes from each category are further split into pan-genome category in **A** or duplication mechanism in **B** and overlaid over their parent category. Outliers (> Q3 + 1.5x IQR) are plotted as dots. Significance testing was performed using a Kruskal-Wallis test with Dunn-Sidak correction. **C**. Histogram showing distribution of positively selected sites per nucleotide split by RLK subfamily. OGs are separated into 50 bins with each bin representing the number of positively selected sites per nucleotide. Values represent the number of OGs found within that bin. Dotted lines represent the number of standard deviations from the mean (red), with black = ±1 standard deviation, orange = +2 standard deviations, and blue = +3 standard deviations. Any OGs with a value greater than +3 standard deviations away were merged into one bin. Most OGs fell within ±1 standard deviation from the mean. **D**-**E**. Boxes show the number of positively selected sites per nucleotide for RLKs separated by duplication mechanism in **D** or pan-genome category in **E**. Boxes from each category are further split into pan-genome category in **D** or duplication mechanism in **E** and overlaid over their parent category. Outliers (> Q3 + 1.5x IQR) are plotted as dots. Significance testing was performed using a Kruskal-Wallis test with Dunn-Sidak correction

Neofunctionalization through positive selection is an important driver of diversification and adaptation [54, 55]. We therefore examined the RLKs for evidence of this process by identifying the occurrences of non-synonymous and synonymous substitutions to determine the average ratio of non-synonymous to synonymous substitutions gene-wide (Fig. 4A, B). We found the number of non-synonymous substitutions scaled in a step-wise fashion based on pan-genome category, with core OGs showing the lowest and cloud OGs showing the highest amounts (Fig. 4A, B). Comparing the observed rates in OGs arising via different duplication mechanisms, both TD and PD have a significantly higher ratio of non-synonymous to synonymous substitutions than other classes (Fig. 4A, B). Of the top 20 genes with the highest ratio of non-syn to syn substitutions, half were un-annotated with the annotated genes spread among different RLK subfamilies (Table 1).

We then examined the rates of positive selection at each site across OGs. To ensure gene length did not bias positive selection acquisition, we corrected by gene length. We then split these values into bins and investigated the distribution of positively selected sites across the RLK subfamilies (Fig. 4C). Overall, the rate of positive selection across subfamilies is within ± 1 standard deviation of the mean for the majority of RLKs (Fig. 4C). The CRK, LRK10L, LRR, MLRR, PR5L, RLCK, SRK, and WAKL subfamilies all contain OGs with a large amount of positive selection greater than 3 standard deviations from the mean (Fig. 4C). Again, the top 20 OGs with the highest rate of positive selection were mostly un-annotated (Table 2).

**Table 2:** Top 20 OGs with highest number of positively selected sites per nucleotide

Most similar gene	OG	PSS per nucleotide^1^	NSS per nucleotide^2^	Family	Pangenome category	Duplication mechanism
AT1G65250	N0.HOG0000002_2	0.146137787	0.096033403	RLCK	Shell	Proximal
AT1G65250	N0.HOG0000002_1	0.143141153	0.11332008	RLCK	Almost Core	Proximal
AT1G35710	N0.HOG0000039_1	0.075114679	0.123853211	LRR	Core	Dispersed
AT1G67470/ZRK12	N0.HOG0001002_1	0.070539419	0.03526971	RLCK	Almost Core	Dispersed
AT4G20450	N0.HOG0000035_1	0.06043956	0.113186813	MLRR	Core	Dispersed
AT4G11460/CRK30	N0.HOG0000604	0.056865465	0.058252427	CRK	Core	Tandem
AT5G59670	N0.HOG0000020_2	0.050561798	0.033707865	MLRR	Shell	Tandem
AT1G65190	N0.HOG0000007_2	0.049528302	0.044811321	RLCK	Shell	Proximal
AT1G66910	N0.HOG0000029	0.049180328	0.052693208	LRK10L	Shell	Tandem
AT1G69730	N0.HOG0000041_1	0.046172539	0.059538275	WAKL	Core	Dispersed
AT1G65250/ZRK14	N0.HOG0000002_3	0.044871795	0.051282051	RLCK	Cloud	Proximal
AT1G67520	N0.HOG0000995	0.040189125	0.027186761	SRK	Almost Core	Dispersed
AT1G65190/ZRK13	N0.HOG0000007_1	0.035629454	0.03087886	RLCK	Almost Core	Dispersed
AT1G21250/WAK1	N0.HOG0000147	0.035326087	0.020380435	WAKL	Core	Tandem
AT1G67000	N0.HOG0000056_1	0.034058657	0.051087985	LRK10L	Core	Proximal
AT1G17910	N0.HOG0000135	0.030864198	0.062962963	WAKL	Core	Dispersed
AT4G04510/CRK38	N0.HOG0000592	0.030769231	0.047692308	CRK	Core	Tandem
AT2G14510	N0.HOG0000012_1	0.029312289	0.065388952	MLRR	Almost Core	Proximal
AT5G38280/PR5K	N0.HOG0000970	0.028694405	0.055954089	PR5L	Almost Core	Proximal
AT4G11900	N0.HOG0000609	0.028669725	0.033256881	SRK	Core	Dispersed

^1^Positively selected sites (PSS)

^2^Negatively selected sites (NSS)

We also wanted to determine if the rate of positive selection varied by pan-genome category or duplication mechanism. OGs that arose through TD have significantly more positive selection than those that arose through DSD or WGD/Segmental duplication, while those that arose through PD have significantly more than DSD only (Fig. 4D). In contrast, OGs within the almost core and shell genomes have significantly more positively selected sites than core and cloud OGs, with cloud OGs having the fewest positively selected sites (Fig. 4E). In fact, core OGs overall had significantly higher positive selection than cloud OGs (Fig. 4E). Overall, OGs that arose through TD tended to have the highest contribution to positive selection within each pan-genome category (Fig. 4E).

### The ECD experiences significantly more positive selection than the KD

**Fig. 5:**
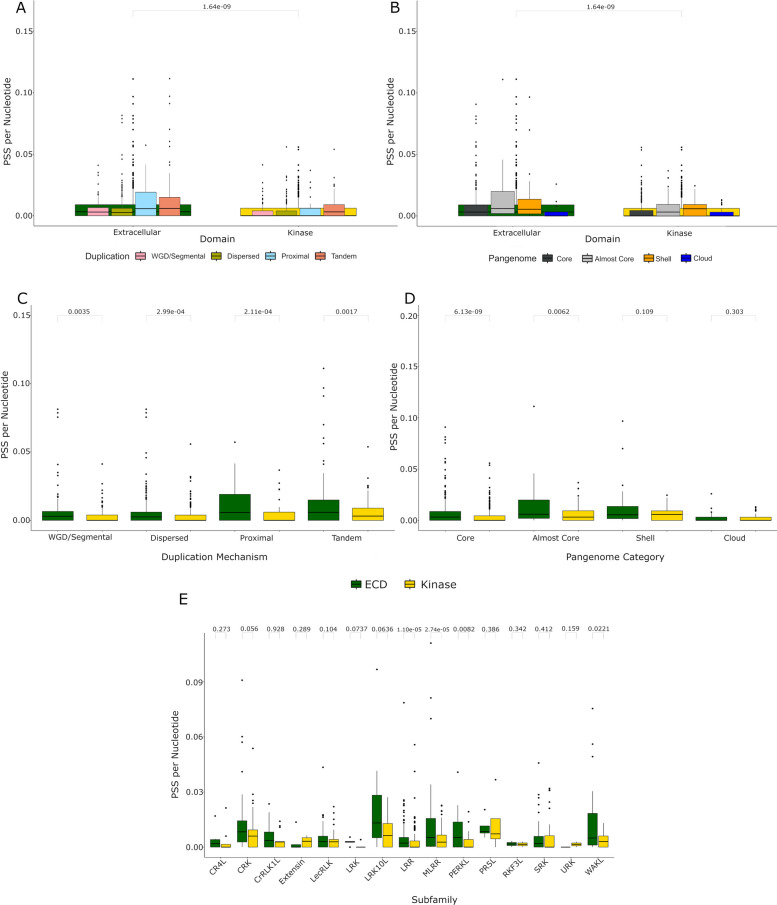
RLK ECDs show higher rates of positive selection than KDs. **A**-**B**. Boxes show the number of positively selected sites per nucleotide split by either the ECD or KD. Boxes for each domain feature are divided into duplication mechanism **A** or pan-genome category **B** and overlaid over the domain box from which they were split. Outliers (> Q3 + 1.5x IQR) are plotted as dots. Significance testing was performed using a Kruskal-Wallis test with Dunn-Sidak correction. **C**-**E**. Number of positively selected sites per nucleotide are compared between the ECD or KD and split by duplication mechanism (**C**), pan-genome category (**D**), or subfamily (**E** Outliers (> Q3 + 1.5x IQR) are plotted as dots. Significance testing was performed using a Kruskal-Wallis test with Dunn-Sidak correction

It has been shown previously that the ECD experiences more diversifying selection than the KD in the members of the LRR-RLK subfamily [56]. We therefore tested whether this phenomenon extends to all RLKs. To do so, we compared the rate of pervasive positive selection in the ECD to the KD across the RLKs. We observed significantly more positive selection in the ECD of RLKs compared to the KD (Fig. 5A, B). This difference is driven primarily by variability between pan-genome categories rather than duplication mechanisms (Fig. 5C, D). The effect was universal across duplication mechanisms, where all had significantly more positive selection in the ECD than the KD (Fig. 5C). OGs within the core and almost core category had the largest differences, with OGs in both containing significantly more positive selection in the ECD than KD (Fig. 5D). Shell and Cloud OGs did not have significantly more positive selection in the ECD over the KD (Fig. 5D). Across all RLK subfamilies, the LRR, MLRR, PERKL, and WAKL subfamilies all have significantly more positive selection in the ECD compared to the KD (Fig. 5E).

### Gene presence/absence variation occurs in clusters

**Fig. 6:**
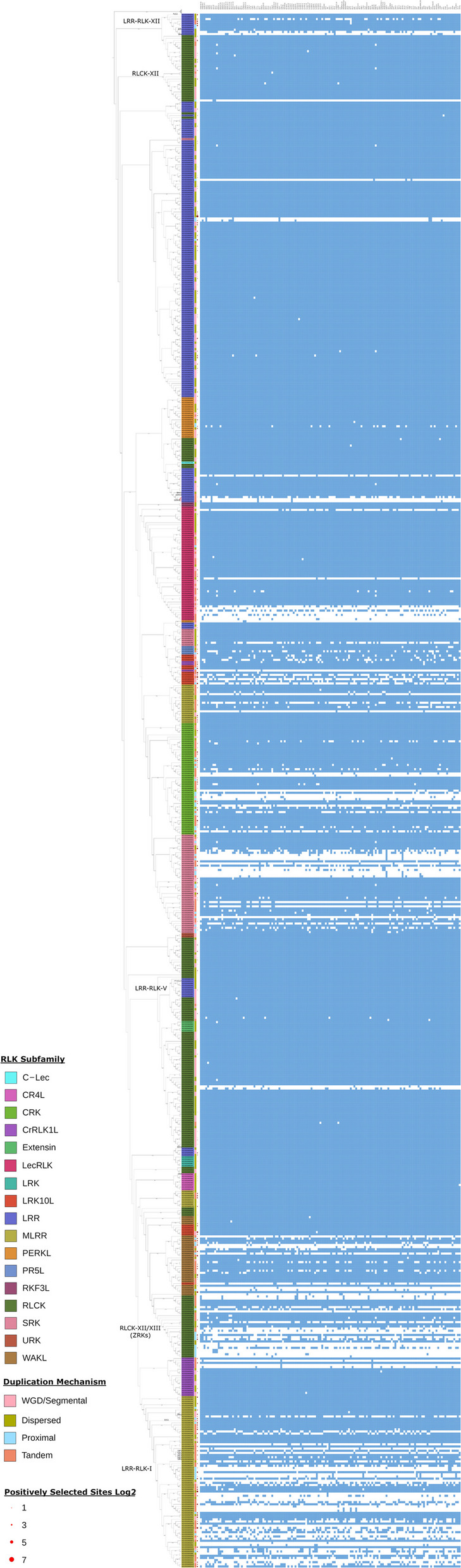
RLK OGs show varying rates of presence/absence variation. Blue squares represent present OGs, white represent absent OGs. Red dots represent the log2 scaled number of positively selected sites found across genes of that OG. OGs are ordered based on the phylogeny inferred from the consensus kinase sequence of each OG. Coloured boxes beside the OG names represent the mechanism of duplication by which the majority of genes within that OG arose. Select gene subfamilies and most-similar annotated genes are labelled

**Fig. 7:**
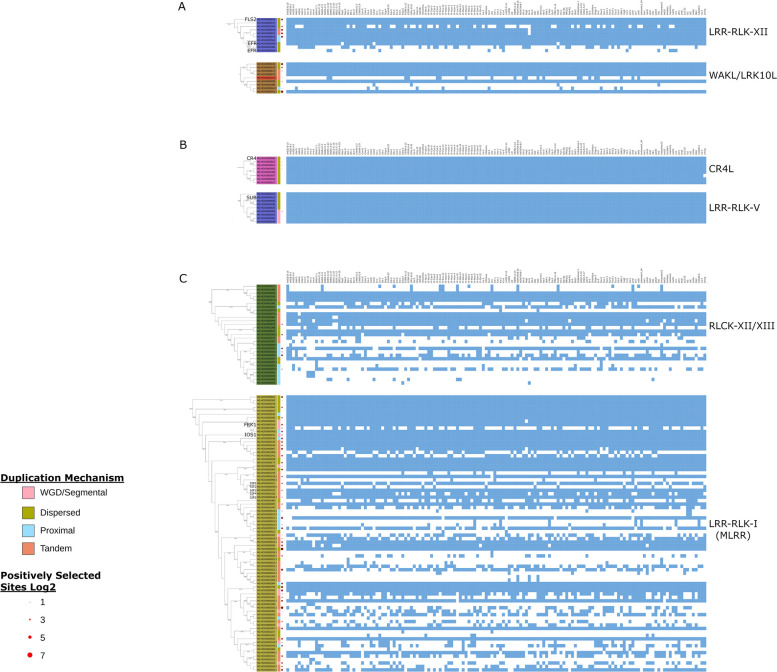
RLK OG adaptive traits comparison across subclades. Blue squares represent present OGs, white represent absent OGs. Red dots represent the log2 scaled number of positively selected sites found across genes of that OG. OGs are ordered based on the phylogeny inferred from the consensus kinase sequence of each OG. Coloured boxes beside the OG names represent the mechanism of duplication by which the majority of genes within that OG arose. Select gene subfamilies and most-similar annotated genes are labelled. Comparisons are between the LRR-RLK-XII subclade and a subclade of the WAKL/LRK10L in **A**, between the CR4L and LRR-RLK-V subclades in **B**, and between the RLCK-XII/XIII and LRR-RLK-I (MLRR) subclades in **C**

Lastly, we examined the gene PAV across the RLK OGs as it is a strong indicator of stress-adaptation [33, 47]. Most OGs do not display high rates of PAV, though there are clear outliers from this phenomenon such as the MLRRs and RLCKs that show extensive PAV (Fig. 6, selected comparisons in Fig. 7). The MLRRs are divided into three main subclades (LRR-RLK-I, LRR-RLK VIII.1, and LRR-RLK VIII.2), with each showing a different PAV profile (Fig. 6). LRR-RLK-I has an almost universally high amount of PAV, LRR-RLK-VIII.1 has little PAV, while LRR-RLK-VIII.2 has selectively high PAV in four OGs (Fig. 6). Similarly, RLCKs show a divergent PAV profile across subclades (Fig. 6). Of the approximately 17 RLCK subclades, most show little to no PAV (Fig. 6). The only outlier is the RLCK subclade XII/XIII which contains a high degree of PAV (Fig. 6, selected comparisons in Fig. 7).

## Discussion

To aid in studies of plant RLK diversity, we have compiled a nearly complete species-wide inventory of RLKs in Arabidopsis. The inventory becomes nearly saturated after the incorporation of only 10–25 of the 146 ecotype genomes used (Fig. 2B). This lack of overall diversity is coupled with little observed variation in the total number of RLKs present in each subfamily across ecotypes (Fig. 1B). At this point, the incorporation of additional genomes results in an average gain of less than one novel OG indicating that the majority of total diversity has been captured and the pan-RLKome is closed (Fig. 2B, C). These observations are quite different from what has been previously reported, and confirmed here, for the Arabidopsis pan-NLRome [47]. The pan-RLKome has both more OGs and saturates much more quickly than the pan-NLRome, indicating a lower level of overall diversity. Assigning the RLK OGs to pan-genome categories indicates a similarly reduced level of overall diversity. Excluding private genes, approximately 80% of RLK OGs are found within the core or almost core genomes, with only ~ 20% assigned to the dispensable genome (Fig. 2G). This again contrasts greatly with the NLRs, which have almost 60% of OGs within the dispensable genome (Fig. 2G) [47]. This is expected, because the NLRs are exclusively stress-related while the RLKs are known to have a broad diversity of functions [1, 27].

In our study, the Arabidopsis ecotypes were sampled from across the species’ natural habitats, including both relicts and non-relicts [57, 58]. The non-relicts are the most widespread and abundant populations throughout the world [57–59]. During the last glacial period, the non-relict progenitor spread throughout Europe and Eastern Asia and intermixed with local relict populations, resulting in the predominance of the non-relicts [58, 60]. At this time, it is likely that we have sampled the majority of the extant diversity across the existing ecotypes of this species, though it is possible that there exist special refugia where relict populations may be present and were untouched by the spread of the non-relicts [58–62]. If such regions exist and are sampled, it would be interesting to see if they contain increased diversity not captured by the ecotypes used in this study.

Different domains and regions of proteins can face different selective pressures that shape their adaptation [47, 56, 63]. We found that the ECD of the RLKs overall is under higher positive selection than the KD, with a significantly higher rate of positively selected sites (PSS) per nucleotide (Fig. 5). This same observation was previously made within the LRR-RLK subfamily [56], but here we show that this is a general trait of RLK subfamilies. In their research, Man and colleagues hypothesized that evidence of escape from adaptive conflict may be a driver of the asymmetric evolution of the ECD and KDs in the LRR-RLKs [56]. In this hypothesis, receptors may gain additional functional capabilities that come at the cost of adaptive conflict where each function is beneficial in conflicting environments [64]. Gene duplication can resolve this conflict through the processes of subfunctionalization and/or neofunctionalization [64]. This provides a possible explanation for why the majority of OGs have significantly more positive selection in the ECD than the KD (Fig. 5C).

However, this pattern is only observed in core or almost core OGs (Fig. 5). Shell and cloud OGs do not have significantly more positive selection in the ECD (Fig. 5). Cloud OGs also have significantly fewer total positively selected sites than core, almost core, and shell OGs (Fig. 4E), yet have a significantly higher ratio of non-syn to syn substitutions gene-wide than any other pan-genome category (Fig. 4B). This may be explained by relaxation of purifying selection around new genes, allowing rapid accumulation of mutations [65]. It is hypothesized that there is a period following duplication where one paralog is free to rapidly accumulate mutations [65]. As cloud OGs tend to be formed through TD/PD (Fig. 3D, E), the reason we may see less positive selection in cloud and shell genomes may be that they have not had enough time for evidence of selection to accumulate. Alternatively, these genes may accumulate changes too rapidly across different ecotypes for selection programs to detect positive selection, or it may be due to a limitation of the methods used here to detect positive selection with low sequence input [66].

As the RLKs are a very large and heterogeneous family, we also divided them into subfamilies for more detailed analysis. While the RLKs as a whole do not show strong evidence of the patterns expected for stress-responsive genes, separating them by subfamilies revealed such evidence. Although no RLK sub-family shows the same proportion of dispensable genes as the NLRs, several do show enrichment. In particular, the LRK10Ls and MLRRs have just under 50% dispensable OGs (Fig. 2E). Many subfamilies also show elevated rates of TD and PD compared to other RLKs, with some approaching the overall proportion observed in the NLRs (Fig. 3A–C). These include the CRK, MLRR, PR5L, SRK, and WAKL subfamilies (Fig. 3C). We also see elevated PAV within specific subclades of the RLK subfamilies (Fig. 6). The RLCK, SRK, MLRR, LecRLK, CRK, and LRK10L subfamilies all have subclades with elevated PAV (Fig. 6). An elevated rate of PAV and reliance on TD or PD for expansion are both characteristics associated with gene subfamilies involved in stress response [47]. Subfamilies with both a high rate of TD/PD and increased PAV may be more likely to have functions in stress adaptation. In contrast, the most frequently observed mechanisms of expansion for the RLCKs were WGD/Segmental duplication (Fig. 3B, C). This is consistent with the finding that WGD/Segmental duplication is over-represented as a mechanism of duplication for genes involved in signal transduction [34].

To more closely investigate the subfamilies that showed increased expansion of the dispensable genome and evidence of stress-related selection, we mapped these data onto the phylogeny of the RLK OGs and included the mechanism of duplication (Figs. 6, 7). This visualization reveals clear regions enriched in PAV not generally seen across entire subfamilies (Figs. 6, 7). Instead, these regions are often limited to one or two monophyletic clades within a given subfamily and are correlated with an increase of TD/PD (Fig. 6). For example, only ~ 27% of the CRK subfamily OGs are present within the dispensable genome, but these genes are closely related and found in two distinct clades with increased rates of PAV (Fig. 6). This is coupled with the observation that the CRK subfamily has the highest proportion of TD among the RLKs, consistent with the CRKs’ known role in stress adaptation (Fig. 3C) [1, 16].

These analyses may be a useful method to better predict RLK subfamily function. To test whether known examples conform to these patterns, we examined three examples—one RLK subfamily known to have stress-related genes, one with known developmental function, and one believed to assist NLRs in effector recognition.

In our first example, the subclade LRR-RLK-XII of the LRR-RLK subfamily contains two well-characterized immune receptors FLAGELLIN SENSITIVE 2 (FLS2) and elongation factor TU receptor (EFR) that may have arisen through DSD (Fig. 6) [67, 68]. FLS2 is most similar to genes in N0.HOG0000818 and has a relatively high amount of positive selection (Figs. 6, 7). Diverging from FLS2 are two other small clades, one containing three genes that have a considerable amount of positively selected sites and have arisen through TD/PD and a second clade containing EFR (Figs. 6, 7). EFR is most similar to genes in N0.HOG0000045_1 and 2 and appears to have duplicated in some ecotypes, but is not found in all (Fig. 6). Similarly, there is a WAKL subclade containing N0.HOG0001074 (AT1G66910), N0.HOG0000299 (AT1G79680), N0.HOG0001098 (most similar to AT5G66790), and N0.HOG0000041_1 and 2 (most similar to AT1G69730) that shows a similar pattern (Figs. 6, 7). The subclade contains four OGs that have arisen through DSD, one through TD, and one through PD (Fig. 6). OG N0.HOG0000299 is a core gene that arose through DSD and has some degree of positive selection (Figs. 6, 7). Interestingly, the most similar gene to N0.HOG0000299 is WALL-ASSOCIATED KINASE-LIKE 10 (Additional file 1: Supplemental Table 2), which has been implicated in biotic stress responses [69]. While the other genes in this subclade have no known function, OG N0.HOG0000041_1 is a core gene that may have arisen through DSD and then further duplicated to form N0.HOG0000041_2 in some ecotypes (Figs. 6, 7).

In the second example, the CR4L subfamily of receptors contains the characterized receptor crinkly4, and its members have been shown to function broadly in development [20]. Within this family, there is a limited amount of positive selection, no genes arose through PD/TD, and all genes are almost universally found in all ecotypes (Figs. 6, 7). The LRR-RLK-V subclade shows a similar pattern to the CR4L subfamily (Figs. 6, 7). The LRR-RLK-V subclade contains the characterized receptor STRUBELLIG, which plays an important role in growth, development, and cellulose deficiency [70].

In our third example, the RLCK subclade XII/XIII of RLCKs (Figs. 6, 7) contains many receptors including the ZED1-related kinases (Figs. 6, 7). These RLCKs function in biotic stress response and act as decoys and effector sensors for the NLR ZAR1 [71, 72]. This subclade has high amounts of PAV, with many of the OGs having arisen through TD or PD (Fig. 6). This pattern of high PAV and TD/PD is similar to that of the NLRs [47]. The MLRR subfamily, specifically the LRR-RLK-I subclade, shares a similar pattern seen in the RLCK family XII/XIII and NLRs, suggesting they may play similar biological roles. While most genes within this subclade do not yet have a known function, the studied members include several involved in stress responses, such as IOS1, FRK1, and the SIFs [73–76]. It is possible that like the RLCK-XII/XIII subclade, the high PAV seen in LRR-RLK-I is due to the rapid gain and loss of genes to respond to a changing biotic stressor.

## Conclusions

The generation of the pan-RLKome in Arabidopsis has allowed us to identify evolutionary patterns hidden by investigating ecotype genomes in isolation. The work here is especially important in highlighting the evolutionary patterns of stress adaptation across the RLKs. With this knowledge in hand, researchers can use these patterns to focus investigations on gene subfamilies and lineages that are more targeted to the process they are investigating.

## Methods

### Gene annotation and protein prediction

Long-read genomes from 146 different ecotypes were retrieved from four different sources: 69 ecotypes from Lian et al. [46], 26 from Kang et al. [44], 44 from Wlodzimierz et al. [45], and 8 from our work in Kileeg et al. [51]. Ecotypes are listed in Additional file 1: Supplemental Table 1. Gene models were predicted using a combination of de novo, transcript, and protein inference data following the protocol of Lian et al. [46]. In short, de novo assembly was performed using Augustus [77, 78], glimmerHMM (v3.0.4) [79], and the GeMoMa pipeline v1.9 [80]. 243 paired-end and single-end RNA sequencing experiments from 20 ecotypes were downloaded from NCBI SRA and used to infer transcripts (Additional file 1: Supplemental Table 3). Adapter presence and read quality were determined using fastqQC (v0.11.9) (https://github.com/s-andrews/FastQC). Reads were trimmed and adapters removed as necessary using trimmomatic (v0.39) [81]. As not all ecotypes have corresponding RNA-seq data, reads from the 243 experiments were pooled into one set. Reads from RNA-seq datasets were aligned to each ecotype genome using Hisat2 (v2.2.1) [82]; transcripts were assembled using Stringtie (v2.2.1) [83] and reconstructed using TransDecoder (v5.7.1) (Haas, BJ. https://github.com/TransDecoder/TransDecoder). Lastly, protein sequences annotated from Araport11 [84] were aligned to each ecotype genome using exonerate v2.4.0 [85] using the protein2genome mode and using liftoff v1.6.3 [86] with settings “-p 8 -copies -sc 0.90 -exclude_partial -a 0.9 -polish.” All modes of evidence were combined using EVidenceModeler (v2.1.0) [87]. Gene prediction annotations were merged, and the longest isoform predicted and extracted as the representative sequence using AGAT (v1.3.3) (https://github.com/NBISweden/AGAT). To fix mis-merged and mis-split genes, the evidence modeler annotations were then compared to those from GeMoMa using Bedtools (v2.30.0) [88]. Where the entire GeMoMa inference was covered by the evidence modeler prediction, the GeMoMa annotation was taken. Evidence modeler inferences not matching or partially matching the GeMoMa annotation were taken instead. GeMoMa inferences not supported by the evidence modeler annotation were discarded. Any incomplete coding sequences or sequences that contained internal stop codons were first reoriented in frame using gffread (v0.12.7) [89] and discarded if they could not be properly recovered.

### Generation of RLK and NLR inventories

CDS and protein sequences were extracted from the entire protein coding set using Seqkit (v2.1.0) [90]. Profile hidden-Markov models for protein kinase domain (PF00069, PF07714) were downloaded from Pfam [91]. Protein sequences from the 146 ecotypes containing a kinase domain hit with an E-value < 0.1 were identified using HMMSearch in HMMER (v3.3.2) [92]. Sequences containing fewer amino acids than the minimum number found in a functional kinase domain (250 amino acids) were removed using Seqkit. All kinase-containing genes were clustered into orthogroups (OGs) using OrthoFinder (v2.5.2) [93]. OGs were sorted into different RLK subfamilies using Arabidopsis RLK annotations as subfamily markers [4, 5]. The Col-0 predictions were used as representative sequences and compared to the Araport11 annotated protein set using BLAST + (v2.11.0) [94]. If an OG did not contain a gene from Col-0, the sequence from the next ecotype in the list was used. Genes were considered part of an RLK family if the best hit had over 80% similarity and 80% coverage to a known reference RLK. OGs were designated as core if 146 ecotypes had a gene present in this OG, almost core if 131–145 ecotypes had a gene present, shell if 30–130 ecotypes contained a gene in that OG, cloud if 2–29 ecotypes contained a gene in a given OG, and private if that gene appeared in only one ecotype. The Col-0 gene most similar to each OG is outlined in Additional file 1: Supplemental Table 2.

Identification of NLRs was done using Resistify (v.1.1.4) [52]. OG inference was performed similarly to the RLKs.

### OG refinement

OGs were split so each gene cluster had no more than one gene from each ecotype. BLAST all-by-all searches for protein sequences in each OG were performed using BLAST + (v2.11.0) [94]. Where multiple genes came from the same ecotype in a given OG, we referred to these as multicopy genes, while genes from ecotypes with only one gene in that OG were referred to as single copy genes. In the cases where an ecotype had multiple genes within an OG, we identified the copy with the highest average similarity score and coverage to the set of single copy genes and assigned that gene to the OG. The remaining multicopy genes were split into a new OG. This process was repeated until no multicopy clusters remained or the only remaining genes came from a single ecotype.

### Rarefaction/accumulation curve analysis

Rarefaction and accumulation curves were generated using homemade scripts in R (https://github.com/MottLab/RLKome_Scripts). For the RLKome accumulation curve, ecotype order was first randomized. The set of OGs where the ecotype had a gene present was collected and counted. This was done for each subsequent ecotype until all 146 ecotypes were analyzed. If a newly added OG had not yet been discovered, it was added to the curve and OG number was counted. This was repeated until all ecotypes had been added to the collection curve. To ensure input order did not bias results, this process was repeated 1000 times. For core RLKome discovery, the initial set of OGs was considered the core RLKome. Each time an added ecotype lacked representation in a given OG, that OG was removed from the total set of core OGs. The average of the 1000 replicates for the RLKome and core RLKome was plotted.

### Identification of gene duplication

Gene association was inferred using a BLASTP all-by-all search for the entire protein set of each ecotype. The top 5 bit-score hits with an e-value < 1e-10 were used. Duplication status was then predicted using MCScanX under default settings [95]. In brief, the MCScanX algorithm works as such: all genes are initially labelled as singleton. Genes with significant BLASTP hits to other genes are labelled dispersed. These are relabelled to tandem if these hits are within 2 genes on the chromosome or proximal if these hits are found within 10 genes on the chromosome. Lastly, any genes with significant hits to another gene are relabelled genome/segmental if duplicates are found to be anchor genes within collinear blocks. The duplication mechanism for each OG as a whole was then assigned based on the most frequently observed duplication mechanism within that OG.

### Selection analysis

Stop codons were removed, and coding sequences for the genes found in each OG were translated, aligned, and then reverse translated using the codon-aware aligner DECIPHER (v2.20.0) [96] in R. Phylogenetic trees were inferred from these alignments using FastTree2 (v2.1.11) [97]. Rates of pervasive positive and negative selection were estimated using the generated codon alignments and trees in FUBAR (v2.1) [98] under default settings. The average rates of non-synonymous and synonymous substitutions at each site were used to calculate the average gene-wide non-synonymous to synonymous substitution rate for each OG. Results were parsed, analyzed, and figures generated using homemade scripts in R (https://github.com/MottLab/RLKome_Scripts).

### Phylogenetic analyses

The ecotype relatedness tree was generated using the single-copy OGs generated from OrthoFinder. For each OG, sequences were aligned to one another using MAFFT (v.7.453) [99] and gene trees inferred using FastTree. All gene trees were fed into ASTRAL (v5.7.8) and ecotype relatedness inferred with 1000 bootstrap replicates [100]. Ecotypes were separated into non-relicts, relicts, and unclear if the admixture groups were not clear. All African and Madeira ecotypes were considered relicts.

To generate the RLK phylogeny, protein sequences from each OG were aligned against the other sequences in that OG using MAFFT and the consensus sequences made by generating an HMM profile of each alignment and then outputting the consensus using the HMMEmit tool in HMMER (v3.3.2) [92]. The protein kinase domains (PF00069, PF07714) from Pfam [91] were identified in each consensus OG sequence using HMMSearch in HMMER (HMMER (v3.3.2) [92]. Each consensus kinase sequence for the OGs was aligned using MAFFT, and a maximum-likelihood phylogeny was constructed using the LG + R9 substitution model found using model finder [101] in IQ-TREE with 1000 bootstrap replicates (v1.6.12) [102]. The phylogeny used a variety of human and Arabidopsis non-RLK kinases as outgroups (Additional file 1: Supplemental Table 4).

All phylogenetic data, including the heatmap, was plotted and visualized using ITOL (v.7) [103].

## Supplementary Information

Additional file 1: Tables S1–S8. Table S1 – Table of ecotypes used and their location of isolation. Table S2 – List of orthogroups used including private genes as well as their closest related Col-0 gene. Table S3 – Accession list for RNAseq datasets used for protein inference. Table S4 – List of outgroup sequence names used for phylogenetic inference. Table S5 – Presence absence counts for orthogroups across each ecotype. Table S6 – Duplication mechanism counts and distribution for each RLK orthogroup. Table S7 – Number of sites under pervasive positive or negative selection across the different RLK orthogroups. Table S8 – Average number of synonymous or non-synonymous substitutions across orthogroups.

Additional file 2: Figure S1.

## Data Availability

All sequences inferred for the 760 OGs have been uploaded to Figshare 10.6084/m9.figshare.29132564.v1. Computational scripts used are uploaded to github repository https://github.com/MottLab/RLKome_Scripts. Details about ecotype collection locations, OG names and information, sequencing reads used for gene prediction, outgroups used for phylogenetic inference, and underlying data for selection analysis, duplication analysis, and presence/absence variation analysis is present in supplemental tables.

## Abbreviations

AtPEP: *Arabidopsis thaliana* Plant elicitor peptides
BAK1: Brassinosteroid insensitive 1 associated receptor-like kinase 1
BIK1: Botrytis-induced kinase 1
B-lectin: Bulb-type lectin
BRI1: Brassinosteroid insensitive 1
CERK1: Chitin elicitor receptor kinase 1
C-lectin: Calcium-dependent lectin
CNV: Copy number variation/variant
CRK: Cysteine-rich repeat receptor-like kinase
CrRLK1L: Catharanthus roseus RLK1-like
DC3000/Pst: *Pseudomonas syringae* Pathovar tomato strain DC3000
DSD: Dispersed duplication
DUF26: Domain of unknown function 26
EFR: EF-Tu receptor
EF-Tu: Elongation factor thermo unstable
EGF: Epidermal growth factor
FLS2: Flagellin-sensitive 2
FRK1: Flagellin-sensitive 2 responsive kinase 1
G-lectin: *Galanthus nivalis* Agglutinin-related lectin
IOS1: Impaired oomycete dusceptibility 1
LecRLK: L-type lectin RLK
L-lectin: Legume-type lectin
LRK10L: LRK10-like RLK
LRR: Leucine-rich repeat OR short form for LRR-RLK
LRR-I: Shortened form of LRR-RLK-I
LRR-RLK: Leucine-rich repeat receptor-like kinase
LRR-VIII.1: Shortened form of LRR-RLK-VIII.1
LRR-VIII.2: Shortened from LRR-RLK-VIII.2
LRR-XI: Shortened form of LRR-RLK XI
LRR-XII: Shortened from LRR-RLK-XII
LYK: Lysin-motif RLK
LysM: Lysin-motif
MAMP: Microbial-associated molecular pattern
MAPK: Mitogen-activated protein kinase
MD/MLD: Malectin domain/malectin-like domain
MLRR: Short form for MLRR-RLK
MLRR-RLK: Malectin-containing LRR-RLK
NLR: Nucleotide-binding leucine-rich repeat receptors
NSS: Negatively selected sites
OG: Orthogroup
PAV: Presence/absence variation
PD: Proximal duplication
PR5L: Pathogenesis related kinase 5-like
PRR: Pattern recognition receptor
PSS: Positively selected sites
PTI: Pattern-triggered immunity
RLK: Receptor-like kinase
ROS: Reactive oxygen species
SIF: Stress-induced factor
TD: Tandem duplication
TE: Transposable element
TIR: Terminal inverted repeat
WAK: Wall-associated kinase
WAKL: Wall-associated kinase-like
WGD: Whole genome duplication

## References

[1] Liu J, Li W, Wu G, Ali K. An update on evolutionary, structural, and functional studies of receptor-like kinases in plants. Front Plant Sci. 2024. 10.3389/fpls.2024.1305599. 38362444 10.3389/fpls.2024.1305599 PMC10868138

[2] Jamieson PA, Shan L, He P. Plant cell surface molecular cypher: receptor-like proteins and their roles in immunity and development. Plant Sci. 2018;274:242–51. 30080610 10.1016/j.plantsci.2018.05.030 PMC6297115

[3] Escocard de Azevedo Manhães AM, Ortiz‐Morea FA, He P, Shan L. Plant plasma membrane‐resident receptors: surveillance for infections and coordination for growth and development. J Integr Plant Biol. 2021;63:79–101. 10.1111/jipb.13051 PMC7855669 33305880

[4] Shiu S-H, Bleecker AB. Receptor-like kinases from Arabidopsis form a monophyletic gene family related to animal receptor kinases. Proc Natl Acad Sci U S A. 2001;98:10763–8. 11526204 10.1073/pnas.181141598 PMC58549

[5] Shiu SH, Karlowski WM, Pan R, Tzeng YH, Mayer KFX, Li WH. Comparative analysis of the receptor-like kinase family in Arabidopsis and rice. Plant Cell. 2004;16:1220–34. 15105442 10.1105/tpc.020834 PMC423211

[6] Liu P-L, Du L, Huang Y, Gao S-M, Yu M. Origin and diversification of leucine-rich repeat receptor-like protein kinase (LRR-RLK) genes in plants. BMC Evol Biol. 2017;17:47. 28173747 10.1186/s12862-017-0891-5 PMC5296948

[7] Yang H, Wang D, Guo L, Pan H, Yvon R, Garman S, et al. Malectin/Malectin-like domain-containing proteins: a repertoire of cell surface molecules with broad functional potential. The Cell Surface. 2021;7: 100056. 34308005 10.1016/j.tcsw.2021.100056 PMC8287233

[8] Dufayard JF, Bettembourg M, Fischer I, Droc G, Guiderdoni E, Périn C, et al. New insights on leucine-rich repeats receptor-like kinase orthologous relationships in angiosperms. Front Plant Sci. 2017;8. 10.3389/fpls.2017.00381 PMC5380761 28424707

[9] Fischer I, Diévart A, Droc G, Dufayard J-F, Chantret N. Evolutionary dynamics of the leucine-rich repeat receptor-like kinase (LRR-RLK) subfamily in angiosperms. Plant Physiol. 2016;170:1595–610. 26773008 10.1104/pp.15.01470 PMC4775120

[10] Yeh YH, Panzeri D, Kadot Y, Huang YC, Huang PY, Tao CN, et al. The arabidopsis malectin-like/LRR-RLK IOS1 is critical for BAK1-dependent and BAK1-independent pattern-triggered immunity. Plant Cell. 2016;28:1701–21. 27317676 10.1105/tpc.16.00313 PMC5077175

[11] Giordano L, Allasia V, Cremades A, Hok S, Panabières F, Bailly-Maître B, et al. A plant receptor domain with functional analogies to animal malectin disables ER stress responses upon infection. iScience. 2022;25: 103877. 35243239 10.1016/j.isci.2022.103877 PMC8861646

[12] Lee HK, Canales Sanchez LE, Bordeleau SJ, Goring DR. *Arabidopsis* leucine-rich repeat malectin receptor–like kinases regulate pollen–stigma interactions. Plant Physiol. 2024;195:343–55. 38270530 10.1093/plphys/kiae038

[13] Franck CM, Westermann J, Boisson-Dernier A. Plant malectin-like receptor kinases: from cell wall integrity to immunity and beyond. Annu Rev Plant Biol. 2018;69 March:301–28. 10.1146/annurev-arplant-042817-040557 29539271

[14] Wu F, Chi Y, Jiang Z, Xu Y, Xie L, Huang F, et al. Hydrogen peroxide sensor HPCA1 is an LRR receptor kinase in Arabidopsis. Nature. 2020;578:577–81. 32076270 10.1038/s41586-020-2032-3

[15] Vaattovaara A, Brandt B, Rajaraman S, Safronov O, Veidenberg A, Luklová M, et al. Mechanistic insights into the evolution of DUF26-containing proteins in land plants. Commun Biol. 2019. 10.1038/s42003-019-0306-9. 30775457 10.1038/s42003-019-0306-9 PMC6368629

[16] Zeiner A, Colina FJ, Citterico M, Wrzaczek M. Cysteine-rich receptor-like protein kinases: their evolution, structure, and roles in stress response and development. J Exp Bot. 2023;74:4910–27. 37345909 10.1093/jxb/erad236

[17] Zhang Z, Huo W, Wang X, Ren Z, Zhao J, Liu Y, et al. Origin, evolution, and diversification of the wall-associated kinase gene family in plants. Plant Cell Rep. 2023;42:1891–906. 37743376 10.1007/s00299-023-03068-9

[18] Feuillet C, Schachermayr G, Keller B. Molecular cloning of a new receptor-like kinase gene encoded at the Lr10 disease resistance locus of wheat. Plant J. 1997;11:45–52. 9025301 10.1046/j.1365-313x.1997.11010045.x

[19] Zhou H, Li S, Deng Z, Wang X, Chen T, Zhang J, et al. Molecular analysis of three new receptor-like kinase genes from hexaploid wheat and evidence for their participation in the wheat hypersensitive response to stripe rust fungus infection. Plant J. 2007;52:420–34. 17764502 10.1111/j.1365-313X.2007.03246.x

[20] Czyzewicz N, Nikonorova N, Meyer MR, Sandal P, Shah S, Vu LD, et al. The growing story of (ARABIDOPSIS) CRINKLY 4. J Exp Bot. 2016;67:4835–47. 27208540 10.1093/jxb/erw192

[21] Dievart A, Gottin C, Périn C, Ranwez V, Chantret N. Origin and diversity of plant receptor-like kinases. Annu Rev Plant Biol. 2020;71:131–56. 32186895 10.1146/annurev-arplant-073019-025927

[22] Invernizzi M, Hanemian M, Keller J, Libourel C, Roby D. PERking up our understanding of the proline-rich extensin-like receptor kinases, a forgotten plant receptor kinase family. New Phytol. 2022;235:875–84. 35451507 10.1111/nph.18166

[23] Bellande K, Bono JJ, Savelli B, Jamet E, Canut H. Plant lectins and lectin receptor-like kinases: how do they sense the outside? Int J Mol Sci. 2017. 10.3390/ijms18061164. 28561754 10.3390/ijms18061164 PMC5485988

[24] De Coninck T, Van Damme EJM. Plant lectins: handymen at the cell surface. The Cell Surface. 2022;8: 100091. 36465479 10.1016/j.tcsw.2022.100091 PMC9713479

[25] Hailemariam S, Liao C-J, Mengiste T. Receptor-like cytoplasmic kinases: orchestrating plant cellular communication. Trends Plant Sci. 2024;29:1113–30. 38816318 10.1016/j.tplants.2024.04.006

[26] Ngou BPM, Ding P, Jones JDG. Thirty years of resistance: zig-zag through the plant immune system. Plant Cell. 2022;34:1447–78. 35167697 10.1093/plcell/koac041 PMC9048904

[27] Jones JDG, Staskawicz BJ, Dangl JL. The plant immune system: from discovery to deployment. Cell. 2024;187:2095–116. 38670067 10.1016/j.cell.2024.03.045

[28] Yuan Y, Bayer PE, Batley J, Edwards D. Current status of structural variation studies in plants. Plant Biotechnol J. 2021;19:2153–63. 34101329 10.1111/pbi.13646 PMC8541774

[29] Jaegle B, Pisupati R, Soto-Jiménez LM, Burns R, Rabanal FA, Nordborg M. Extensive sequence duplication in Arabidopsis revealed by pseudo-heterozygosity. Genome Biol. 2023;24:44. 36895055 10.1186/s13059-023-02875-3 PMC9999624

[30] Li H, Wang S, Chai S, Yang Z, Zhang Q, Xin H, et al. Graph-based pan-genome reveals structural and sequence variations related to agronomic traits and domestication in cucumber. Nat Commun. 2022;13:682. 35115520 10.1038/s41467-022-28362-0 PMC8813957

[31] Panchy N, Lehti-Shiu M, Shiu S-H. Evolution of gene duplication in plants. Plant Physiol. 2016;171:2294–316. 27288366 10.1104/pp.16.00523 PMC4972278

[32] Picart-Picolo A, Grob S, Picault N, Franek M, Llauro C, Halter T, et al. Large tandem duplications affect gene expression, 3d organization, and plant–pathogen response. Genome Res. 2020;30:1583–92. 33033057 10.1101/gr.261586.120 PMC7605254

[33] Gabur I, Chawla HS, Lopisso DT, von Tiedemann A, Snowdon RJ, Obermeier C. Gene presence-absence variation associates with quantitative *Verticillium longisporum* disease resistance in *Brassica napus*. Sci Rep. 2020;10:1–11. 32139810 10.1038/s41598-020-61228-3 PMC7057980

[34] Qiao X, Li Q, Yin H, Qi K, Li L, Wang R, et al. Gene duplication and evolution in recurring polyploidization-diploidization cycles in plants. Genome Biol. 2019;20:1–23. 30791939 10.1186/s13059-019-1650-2 PMC6383267

[35] Brockhurst MA, Harrison E, Hall JPJ, Richards T, McNally A, MacLean C. The ecology and evolution of pangenomes. Curr Biol. 2019;29:R1094-103. 31639358 10.1016/j.cub.2019.08.012

[36] Gao L, Gonda I, Sun H, Ma Q, Bao K, Tieman DM, et al. The tomato pan-genome uncovers new genes and a rare allele regulating fruit flavor. Nat Genet. 2019;51:1044–51. 31086351 10.1038/s41588-019-0410-2

[37] Golicz AA, Bayer PE, Barker GC, Edger PP, Kim H, Martinez PA, et al. The pangenome of an agronomically important crop plant *Brassica oleracea*. Nat Commun. 2016;7:13390. 27834372 10.1038/ncomms13390 PMC5114598

[38] Hübner S, Bercovich N, Todesco M, Mandel JR, Odenheimer J, Ziegler E, et al. Sunflower pan-genome analysis shows that hybridization altered gene content and disease resistance. Nat Plants. 2018;5:54–62. 30598532 10.1038/s41477-018-0329-0

[39] Jayakodi M, Padmarasu S, Haberer G, Bonthala VS, Gundlach H, Monat C, et al. The barley pan-genome reveals the hidden legacy of mutation breeding. Nature. 2020;588:284–9. 33239781 10.1038/s41586-020-2947-8 PMC7759462

[40] Zhang F, Xue H, Dong X, Li M, Zheng X, Li Z, et al. Long-read sequencing of 111 rice genomes reveals significantly larger pan-genomes. Genome Res. 2022. 10.1101/gr.276015.121. 35396275 10.1101/gr.276015.121 PMC9104699

[41] Walkowiak S, Gao L, Monat C, Haberer G, Kassa MT, Brinton J, et al. Multiple wheat genomes reveal global variation in modern breeding. Nature. 2020;588:277–83. 33239791 10.1038/s41586-020-2961-x PMC7759465

[42] Li Y, Zhou G, Ma J, Jiang W, Jin L, Zhang Z, et al. De novo assembly of soybean wild relatives for pan-genome analysis of diversity and agronomic traits. Nat Biotechnol. 2014;32:1045–52. 25218520 10.1038/nbt.2979

[43] Jiao W-B, Schneeberger K. Chromosome-level assemblies of multiple *Arabidopsis* genomes reveal hotspots of rearrangements with altered evolutionary dynamics. Nat Commun. 2020;11:989. 32080174 10.1038/s41467-020-14779-y PMC7033125

[44] Kang M, Wu H, Liu H, Liu W, Zhu M, Han Y, et al. The pan-genome and local adaptation of *Arabidopsis thaliana*. Nat Commun. 2023;14:6259. 37802986 10.1038/s41467-023-42029-4 PMC10558531

[45] Wlodzimierz P, Rabanal FA, Burns R, Naish M, Primetis E, Scott A, et al. Cycles of satellite and transposon evolution in Arabidopsis centromeres. Nature. 2023;618:557–65. 37198485 10.1038/s41586-023-06062-z

[46] Lian Q, Huettel B, Walkemeier B, Mayjonade B, Lopez-Roques C, Gil L, et al. A pan-genome of 69 *Arabidopsis thaliana* accessions reveals a conserved genome structure throughout the global species range. Nat Genet. 2024;56:982–91. 38605175 10.1038/s41588-024-01715-9 PMC11096106

[47] Van de Weyer A-L, Monteiro F, Furzer OJ, Nishimura MT, Cevik V, Witek K, et al. A species-wide inventory of NLR genes and alleles in *Arabidopsis thaliana*. Cell. 2019;178:1260-1272.e14. 31442410 10.1016/j.cell.2019.07.038 PMC6709784

[48] Bayer PE, Golicz AA, Tirnaz S, Chan CKK, Edwards D, Batley J. Variation in abundance of predicted resistance genes in the *Brassica oleracea* pangenome. Plant Biotechnol J. 2019;17:789–800. 30230187 10.1111/pbi.13015 PMC6419861

[49] Golicz AA, Bayer PE, Bhalla PL, Batley J, Edwards D. Pangenomics comes of age: from bacteria to plant and animal applications. Trends Genet. 2020;36:132–45. 31882191 10.1016/j.tig.2019.11.006

[50] Della Coletta R, Qiu Y, Ou S, Hufford MB, Hirsch CN. How the pan-genome is changing crop genomics and improvement. Genome Biol. 2021;22:1–19. 33397434 10.1186/s13059-020-02224-8 PMC7780660

[51] Kileeg Z, Wang P, Mott GA. Chromosome-scale assembly and annotation of eight *Arabidopsis thaliana* ecotypes. Genome Biol Evol. 2024. 10.1093/gbe/evae169. 39101619 10.1093/gbe/evae169 PMC11327923

[52] Smith M, Jones JT, Hein I. Resistify: a novel NLR classifier that reveals helitron-associated NLR expansion in Solanaceae. Bioinform Biol Insights. 2025. 10.1177/11779322241308944. 39845701 10.1177/11779322241308944 PMC11752215

[53] Birchler JA, Yang H. The multiple fates of gene duplications: deletion, hypofunctionalization, subfunctionalization, neofunctionalization, dosage balance constraints, and neutral variation. Plant Cell. 2022;34:2466–74. 35253876 10.1093/plcell/koac076 PMC9252495

[54] Beisswanger S, Stephan W. Evidence that strong positive selection drives neofunctionalization in the tandemly duplicated polyhomeotic genes in *Drosophila*. Proc Natl Acad Sci USA. 2008;105:5447–52. 18381818 10.1073/pnas.0710892105 PMC2291077

[55] Persi E, Wolf YI, Koonin EV. Positive and strongly relaxed purifying selection drive the evolution of repeats in proteins. Nat Commun. 2016;7:13570. 27857066 10.1038/ncomms13570 PMC5120217

[56] Man J, Harrington TA, Lally K, Bartlett ME. Asymmetric evolution of protein domains in the leucine-rich repeat receptor-like kinase family of plant signaling proteins. Mol Biol Evol. 2023. 10.1093/molbev/msad220. 37787619 10.1093/molbev/msad220 PMC10588794

[57] Alonso-Blanco C, Andrade J, Becker C, Bemm F, Bergelson J, Borgwardt KM, et al. 1,135 genomes reveal the global pattern of polymorphism in *Arabidopsis thaliana*. Cell. 2016;166:481–91. 27293186 10.1016/j.cell.2016.05.063 PMC4949382

[58] Fulgione A, Hancock AM. Archaic lineages broaden our view on the history of *Arabidopsis thaliana*. New Phytol. 2018;219:1194–8. 29862511 10.1111/nph.15244

[59] Durvasula A, Fulgione A, Gutaker RM, Alacakaptan SI, Flood PJ, Neto C, et al. African genomes illuminate the early history and transition to selfing in *Arabidopsis thaliana*. Proc Natl Acad Sci U S A. 2017;114:5213–8. 28473417 10.1073/pnas.1616736114 PMC5441814

[60] Lee C-R, Svardal H, Farlow A, Exposito-Alonso M, Ding W, Novikova P, et al. On the post-glacial spread of human commensal *Arabidopsis thaliana*. Nat Commun. 2017;8:14458. 28181519 10.1038/ncomms14458 PMC5309843

[61] Fulgione A, Koornneef M, Roux F, Hermisson J, Hancock AM. Madeiran *arabidopsis thaliana* reveals ancient long-range colonization and clarifies demography in Eurasia. Mol Biol Evol. 2018;35:564–74. 29216397 10.1093/molbev/msx300 PMC5850838

[62] Zeng L, Gu Z, Xu M, Zhao N, Zhu W, Yonezawa T, et al. Discovery of a high-altitude ecotype and ancient lineage of *Arabidopsis thaliana* from Tibet. Sci Bull. 2017;62:1628–30. 10.1016/j.scib.2017.10.007 36659379

[63] Zhang XS, Choi JH, Heinz J, Chetty CS. Domain-specific positive selection contributes to the evolution of *Arabidopsis* leucine-rich repeat receptor-like kinase (LRR RLK) genes. J Mol Evol. 2006;63:612–21. 17031460 10.1007/s00239-005-0187-z

[64] Sikosek T, Chan HS, Bornberg-Bauer E. Escape from adaptive conflict follows from weak functional trade-offs and mutational robustness. Proc Natl Acad Sci USA. 2012;109:14888–93. 22927372 10.1073/pnas.1115620109 PMC3443171

[65] Pegueroles C, Laurie S, Albà MM. Accelerated evolution after gene duplication: a time-dependent process affecting just one copy. Mol Biol Evol. 2013;30:1830–42. 23625888 10.1093/molbev/mst083

[66] Poon AFY, Frost SDW, Pond SLK. Detecting signatures of selection from DNA sequences using Datamonkey. 2009. p. 163–83. 10.1007/978-1-59745-251-9_8 19378144

[67] Chinchilla D, Bauer Z, Regenass M, Boller T, Felix G. The Arabidopsis receptor kinase FLS2 binds flg22 and determines the specificity of flagellin perception. Plant Cell. 2006;18:465–76. 16377758 10.1105/tpc.105.036574 PMC1356552

[68] Zipfel C, Kunze G, Chinchilla D, Caniard A, Jones JDG, Boller T, et al. Perception of the bacterial PAMP EF-Tu by the receptor EFR restricts *Agrobacterium*-mediated transformation. Cell. 2006;125:749–60. 16713565 10.1016/j.cell.2006.03.037

[69] Meier S, Ruzvidzo O, Morse M, Donaldson L, Kwezi L, Gehring C. The *Arabidopsis* wall associated kinase-like 10 gene encodes a functional guanylyl cyclase and is co-expressed with pathogen defense related genes. PLoS One. 2010;5: e8904. 20126659 10.1371/journal.pone.0008904 PMC2811198

[70] Eyüboglu B, Pfister K, Haberer G, Chevalier D, Fuchs A, Mayer KFX, et al. Molecular characterisation of the Strubbelig-receptor family of genes encoding putative leucine-rich repeat receptor-like kinases in *Arabidopsis thaliana*. BMC Plant Biol. 2007;7:1–24. 17397538 10.1186/1471-2229-7-16 PMC1855328

[71] Martel A, Laflamme B, Seto D, Bastedo DP, Dillon MM, Almeida RND, et al. Immunodiversity of the Arabidopsis ZAR1 NLR is conveyed by receptor-like cytoplasmic kinase sensors. Front Plant Sci. 2020. 10.3389/fpls.2020.01290. 32983191 10.3389/fpls.2020.01290 PMC7475706

[72] Seto D, Laflamme B, Guttman DS, Desveaux D. The Arabidopsis ZED1-related kinase genomic cluster is specifically required for effector-triggered immunity. Plant Physiol. 2020;184:1635–9. 33037126 10.1104/pp.20.00447 PMC7723123

[73] Hok S, Allasia V, Andrio E, Naessens E, Ribes E, Panabières F, et al. The receptor kinase impaired oomycete susceptibility1 a ttenuates abscisic acid responses in Arabidopsis. Plant Physiol. 2014;166:1506–18. 25274985 10.1104/pp.114.248518 PMC4226379

[74] Hsu FC, Chou MY, Chou SJ, Li YR, Peng HP, Shih MC. Submergence confers immunity mediated by the WRKY22 transcription factor in Arabidopsis. Plant Cell. 2013;25:2699–713. 23897923 10.1105/tpc.113.114447 PMC3753392

[75] Pagnussat GC, Yu H-J, Ngo QA, Rajani S, Mayalagu S, Johnson CS, et al. Genetic and molecular identification of genes required for female gametophyte development and function in Arabidopsis. Development. 2005;132:603–14. 15634699 10.1242/dev.01595

[76] Yuan N, Yuan S, Li Z, Zhou M, Wu P, Hu Q, et al. Stress induced factor 2, a leucine-rich repeat kinase regulates basal plant pathogen defense. Plant Physiol. 2018;176:3062–80. 29463771 10.1104/pp.17.01266 PMC5884590

[77] Stanke M, Keller O, Gunduz I, Hayes A, Waack S, Morgenstern B. AUGUSTUS: ab initio prediction of alternative transcripts. Nucleic Acids Res. 2006;34 Web Server:W435–9. 10.1093/nar/gkl200 PMC1538822 16845043

[78] Ter-Hovhannisyan V, Lomsadze A, Chernoff YO, Borodovsky M. Gene prediction in novel fungal genomes using an ab initio algorithm with unsupervised training. Genome Res. 2008;18:1979–90. 18757608 10.1101/gr.081612.108 PMC2593577

[79] Majoros WH, Pertea M, Salzberg SL. Tigrscan and GlimmerHMM: two open source ab initio eukaryotic gene-finders. Bioinformatics. 2004;20:2878–9. 15145805 10.1093/bioinformatics/bth315

[80] Keilwagen J, Hartung F, Grau J. GeMoMa: Homology-based gene prediction utilizing intron position conservation and RNA-seq data. Methods Mol Biol. 2019;1962:161–77. 31020559 10.1007/978-1-4939-9173-0_9

[81] Bolger AM, Lohse M, Usadel B. Trimmomatic: a flexible trimmer for Illumina sequence data. Bioinformatics. 2014;30:2114–20. 24695404 10.1093/bioinformatics/btu170 PMC4103590

[82] Kim D, Paggi JM, Park C, Bennett C, Salzberg SL. Graph-based genome alignment and genotyping with HISAT2 and HISAT-genotype. Nat Biotechnol. 2019;37:907–15. 31375807 10.1038/s41587-019-0201-4 PMC7605509

[83] Pertea M, Pertea GM, Antonescu CM, Chang T-C, Mendell JT, Salzberg SL. Stringtie enables improved reconstruction of a transcriptome from RNA-seq reads. Nat Biotechnol. 2015;33:290–5. 25690850 10.1038/nbt.3122 PMC4643835

[84] Cheng C-YY, Krishnakumar V, Chan AP, Thibaud-Nissen F, Schobel S, Town CD. Araport11: a complete reannotation of the Arabidopsis thaliana reference genome. Plant J. 2017;89:789–804. 27862469 10.1111/tpj.13415

[85] Slater GSC, Birney E. Automated generation of heuristics for biological sequence comparison. BMC Bioinformatics. 2005;6: 31. 15713233 10.1186/1471-2105-6-31 PMC553969

[86] Shumate A, Salzberg SL. Liftoff: accurate mapping of gene annotations. Bioinformatics. 2021;37:1639–43. 33320174 10.1093/bioinformatics/btaa1016 PMC8289374

[87] Haas BJ, Salzberg SL, Zhu W, Pertea M, Allen JE, Orvis J, et al. Automated eukaryotic gene structure annotation using EVidenceModeler and the program to assemble spliced alignments. Genome Biol. 2008;9: R7. 18190707 10.1186/gb-2008-9-1-r7 PMC2395244

[88] Quinlan AR, Hall IM. BEDTools: a flexible suite of utilities for comparing genomic features. Bioinformatics. 2010;26:841–2. 20110278 10.1093/bioinformatics/btq033 PMC2832824

[89] Pertea G, Pertea M. GFF Utilities: GffRead and GffCompare. F1000Res. 2020;9:304. 10.12688/f1000research.23297.1 PMC7222033 32489650

[90] Shen W, Le S, Li Y, Hu F. SeqKit: a cross-platform and ultrafast toolkit for FASTA/Q file manipulation. PLoS One. 2016;11: e0163962. 27706213 10.1371/journal.pone.0163962 PMC5051824

[91] Mistry J, Chuguransky S, Williams L, Qureshi M, Salazar GA, Sonnhammer ELL, et al. Pfam: the protein families database in 2021. Nucleic Acids Res. 2021;49:D412–9. 33125078 10.1093/nar/gkaa913 PMC7779014

[92] Eddy SR. Accelerated profile HMM searches. PLoS Comput Biol. 2011. 10.1371/journal.pcbi.1002195. 22039361 10.1371/journal.pcbi.1002195 PMC3197634

[93] Emms DM, Kelly S. OrthoFinder: phylogenetic orthology inference for comparative genomics. Genome Biol. 2019;20:1–14. 31727128 10.1186/s13059-019-1832-y PMC6857279

[94] Camacho C, Coulouris G, Avagyan V, Ma N, Papadopoulos J, Bealer K, et al. BLAST+: architecture and applications. BMC Bioinformatics. 2009;10:1–9. 20003500 10.1186/1471-2105-10-421 PMC2803857

[95] Wang Y, Tang H, DeBarry JD, Tan X, Li J, Wang X, et al. MCScanX: a toolkit for detection and evolutionary analysis of gene synteny and collinearity. Nucleic Acids Res. 2012;40: e49–e49. 22217600 10.1093/nar/gkr1293 PMC3326336

[96] Wright ES. Using DECIPHER v2.0 to analyze big biological sequence data in R. R Journal. 2016;8:352–9.

[97] Price MN, Dehal PS, Arkin AP. Fasttree 2 – approximately maximum-likelihood trees for large alignments. PLoS One. 2010;5: e9490. 20224823 10.1371/journal.pone.0009490 PMC2835736

[98] Murrell B, Moola S, Mabona A, Weighill T, Sheward D, Kosakovsky Pond SL, et al. FUBAR: a fast, unconstrained bayesian approximation for inferring selection. Mol Biol Evol. 2013;30:1196–205. 23420840 10.1093/molbev/mst030 PMC3670733

[99] Katoh K, Standley DM. MAFFT multiple sequence alignment software version 7: improvements in performance and usability. Mol Biol Evol. 2013;30:772–80. 23329690 10.1093/molbev/mst010 PMC3603318

[100] Zhang C, Rabiee M, Sayyari E, Mirarab S. ASTRAL-III: polynomial time species tree reconstruction from partially resolved gene trees. BMC Bioinformatics. 2018;19(S6): 153. 29745866 10.1186/s12859-018-2129-y PMC5998893

[101] Kalyaanamoorthy S, Minh BQ, Wong TKF, von Haeseler A, Jermiin LS. Modelfinder: fast model selection for accurate phylogenetic estimates. Nat Methods. 2017;14:587–9. 28481363 10.1038/nmeth.4285 PMC5453245

[102] Nguyen L-T, Schmidt HA, von Haeseler A, Minh BQ. IQ-TREE: a fast and effective stochastic algorithm for estimating maximum-likelihood phylogenies. Mol Biol Evol. 2015;32:268–74. 25371430 10.1093/molbev/msu300 PMC4271533

[103] Letunic I, Bork P. Interactive tree of life (iTOL) v6: recent updates to the phylogenetic tree display and annotation tool. Nucleic Acids Res. 2024;52:W78-82. 38613393 10.1093/nar/gkae268 PMC11223838

