# Correction: A species-wide inventory of receptor-like kinases in *Arabidopsis thaliana*

**DOI:** 10.1186/s12915-025-02493-4

**Published:** 2025-12-17

**Authors:** Zachary Kileeg, G. Adam Mott

**Affiliations:** 1 Department of Biological Sciences, University of Toronto - Scarborough, Toronto, Canada; 2 Department of Cell and Systems Biology, University of Toronto, Toronto, Canada; 3 Centre for the Analysis of Genome Evolution & Function, University of Toronto, Toronto, Canada

**Correction: BMC Biol 23, 266 (2025)**

**https://doi.org/10.1186/s12915-025-02364-y**

The original article [1], has been updated to restore the captions for Figures 1–5 which had each erroneously been omitted.
