# Supplementary material for: A species-wide inventory of receptor-like kinases in Arabidopsis thaliana
Source: BMC Biol. 2025 Aug 26;23:266. doi: 10.1186/s12915-025-02364-y (PMC12382012; doi:10.1186/s12915-025-02364-y)
Supplement: Supplementary file 2 — Additional file 2: Figure S1. [file 12915_2025_2364_MOESM2_ESM.pdf]

Figure S1

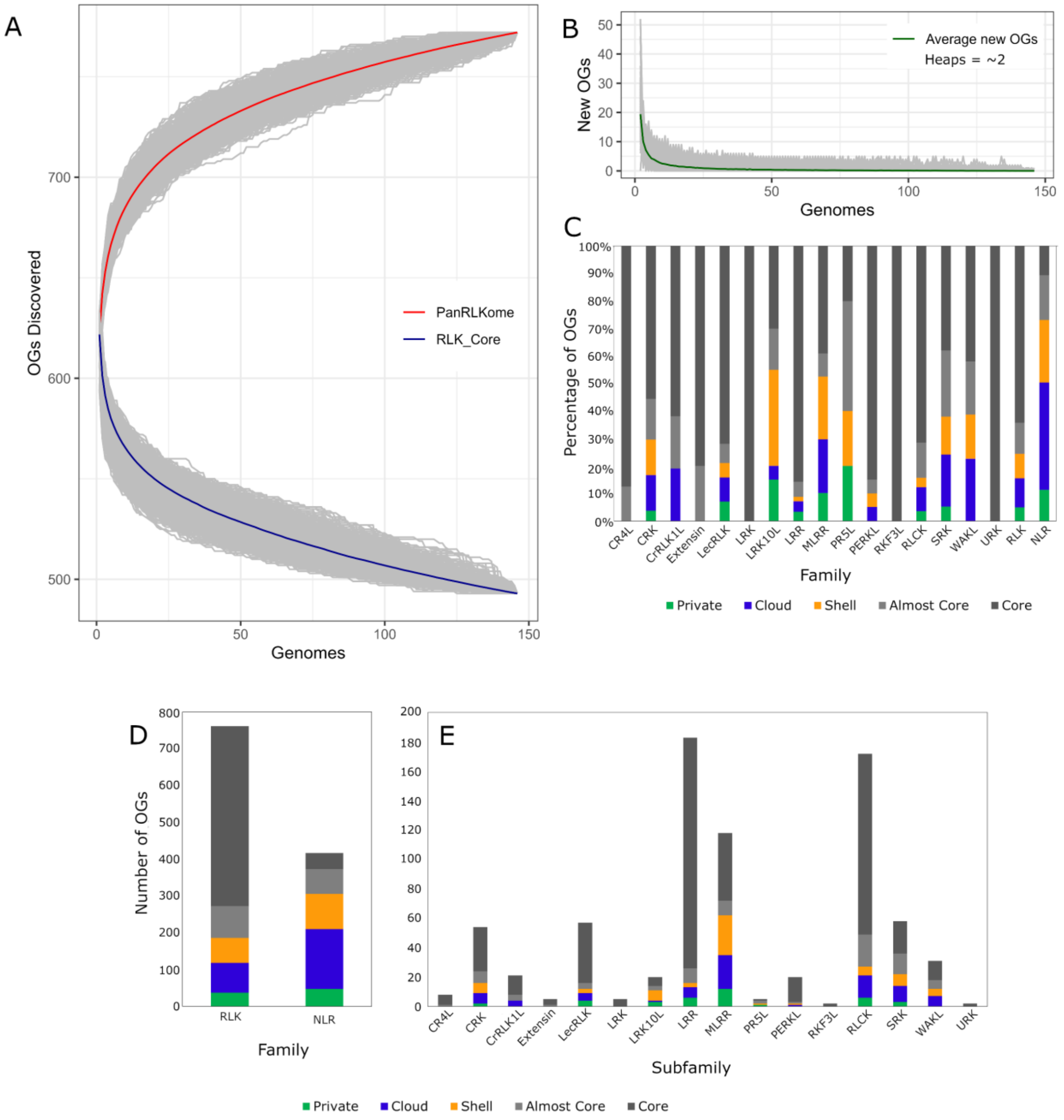

Fig. S1: Orthogroup distribution and saturation including private genes.

A-B. Discovery of novel OGs and pan-RLKome Saturation. Rarefaction **B** and collector's **C** curves show average number of core OGs and newly discovered OGs per ecotype added

respectively. Order of ecotype addition for both analyses was randomized 1000 times and averages plotted.

C-E. RLKs were split into counts by family **C**, by RLK subfamily **D**, or by proportion of RLKs in **E**. In **C** and **D**, the total number of OGs found in each subfamily were coloured by pan-genome category and counted for each. In **E**, the proportion of OGs found in each pan-genome category was calculated for each subfamily. The NLRs were added as a benchmark to both **C** and **E**.
